# Exact and heuristic algorithms for Space Information Flow

**DOI:** 10.1371/journal.pone.0193350

**Published:** 2018-03-27

**Authors:** Alfred Uwitonze, Jiaqing Huang, Yuanqing Ye, Wenqing Cheng, Zongpeng Li

**Affiliations:** 1 School of Electronic Information and Communications, Huazhong University of Science and Technology, Wuhan, 430074, P. R. China; 2 College of Science and Technology, University of Rwanda, Kigali, P. O. BOX 3900, Rwanda; 3 Department of Electrical and Computer Engineering, Carnegie Mellon University, Pittsburgh, Pennsylvania, United States of America; 4 Department of Computer Science, University of Calgary, Calgary, Canada; Universidad Nacional de Mar del Plata, ARGENTINA

## Abstract

*Space Information Flow* (SIF) is a new promising research area that studies network coding in geometric *space*, such as Euclidean space. The design of algorithms that compute the optimal SIF solutions remains one of the key open problems in SIF. This work proposes the first exact SIF algorithm and a heuristic SIF algorithm that compute min-cost multicast network coding for *N* (*N* ≥ 3) given terminal nodes in 2-D Euclidean space. Furthermore, we find that the Butterfly network in Euclidean space is the second example besides the Pentagram network where SIF is strictly better than Euclidean Steiner minimal tree. The exact algorithm design is based on two key techniques: Delaunay triangulation and linear programming. Delaunay triangulation technique helps to find practically good candidate relay nodes, after which a min-cost multicast linear programming model is solved over the terminal nodes and the candidate relay nodes, to compute the optimal multicast network topology, including the optimal relay nodes selected by linear programming from all the candidate relay nodes and the flow rates on the connection links. The heuristic algorithm design is also based on Delaunay triangulation and linear programming techniques. The exact algorithm can achieve the optimal SIF solution with an exponential computational complexity, while the heuristic algorithm can achieve the sub-optimal SIF solution with a polynomial computational complexity. We prove the correctness of the exact SIF algorithm. The simulation results show the effectiveness of the heuristic SIF algorithm.

## Introduction

**Fig 1:**
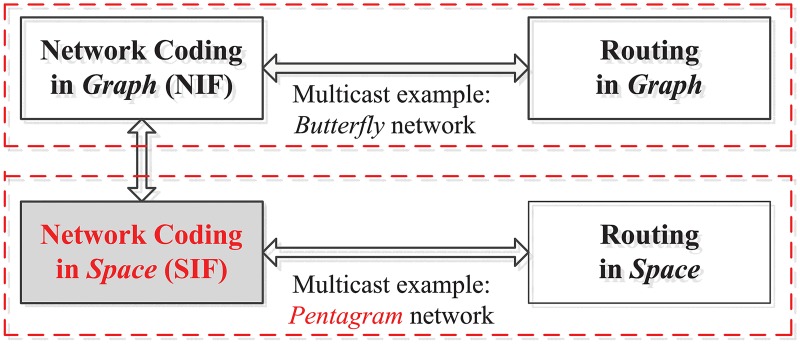
Network coding in space (SIF).

Network Information Flow (NIF) [1], proposed in 2000, studies *network coding in graphs*. Comparatively to NIF, *Space Information Flow* (SIF) [2, 3] studies *network coding in geometric space* (See Fig 1) and it was proposed in 2011. Network coding has been proved to be an effective technology to solve both NIF and SIF problems, and makes it possible to achieve the maximum throughput of a network and to reduce the complexity of computing the optimal transmission scheme [4]. Although both SIF and NIF use network coding as their fundamental technique to transmit information, SIF allows additional set of relay nodes to connect a given set of terminal nodes at any location within the network, something which is not accepted by NIF. In addition, while NIF combines together information theory and graph theory, SIF further brings geometry into the mix. SIF is also viewed as a generalization of Euclidean Steiner Minimal Tree (ESMT) problem by introducing network coding [5]. The ESMT is commonly known as routing in space [6]. SIF is also different from Minimum Spanning Tree (MST) in a way that MST interconnects all the terminal nodes of a given set by a network of direct terminal-to-terminal links with the smallest possible total length, without any additional relay nodes [7], while additional relay nodes are required in SIF [2, 3].

In the SIF model, we are given a set of terminal nodes, with unicast, multicast or broadcast communication demands among them [5]. SIF aims to minimize the total bandwidth-distance sum-product (‘network volume’) in geometric space with a certain throughput requirement, allowing network coding to be used and additional relay nodes to be inserted to connect a given set of terminal nodes, while sustaining end-to-end communication demands among terminals at known coordinates [2]. SIF also opens the door to geometric approaches for studying network information flow problems.

**Fig 2:**
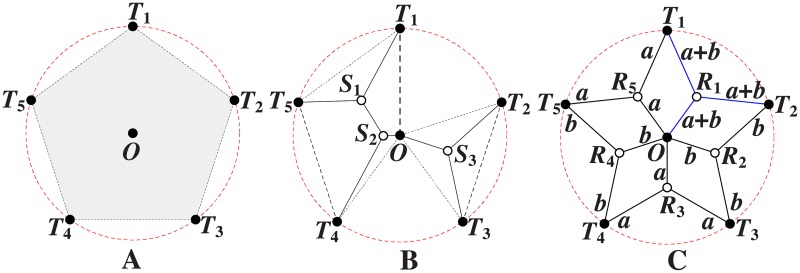
The *Pentagram* example to illustrate a single multicast Space Information Flow problem in 2-D Euclidean space. (A) Six terminal nodes. (B) Euclidean Steiner Minimal Tree (cost = 4.6400/bit). (C) Minimum multicast flow with network coding in space (cost = 4.5677/bit).

The *Pentagram* example [8] illustrated in Fig 2 is the first single multicast example to demonstrate that the performance of SIF can be strictly better than that of ESMT, with the cost advantage [9] being strictly greater than 1, where the cost advantage is defined as the ratio of minimum cost necessary for achieving a target throughput by routing over that of network coding.

We briefly explain the *Pentagram* example as follows: Fig 2(A) shows a single multicast version of the space information flow problem with six terminal nodes in 2-D Euclidean space, where five nodes (*T_1_* to *T_5_*) are evenly distributed along a circle and form a regular pentagon centered at node *O*. The radius of the circumscribed circle is 1. The node *O* is chosen as the multicast source, with the other five being receivers. All the messages to be transmitted are available for the source node *O*, while they are demanded by each sink node (*T_1_* to *T_5_*). The goal is to compare ESMT and SIF, using the *cost* as the comparison metric. The optimal solution based on ESMT can be computed [10, 11] and the cost is 4.6400/bit, as depicted by Fig 2(B). Three Steiner nodes (*S_1_* to *S_3_*) are inserted to connect the terminals, each adjacent to three links that form three angles of 120°. Fig 2(C) shows the optimal solution with SIF, where the total distance is 9.1354, while every sink receives 2 bits. The normalized cost is 9.1354/2 = 4.5677/bit. Five relay nodes (*R_1_* to *R_5_*) are inserted to connect the terminals, each adjacent to three links that form three angles of 120°. The *cost advantage* of the Pentagram example is 4.6400/4.5677≈1.0158>1. Despite its small value, we emphasize that the gap between the two optimal costs reveals that multicast with network coding in space is fundamentally a different problem from geometric ESMT, with a different problem structure, and probably a different computational complexity.

Regarding the applications of SIF, Uwitonze *et al*. [12] proposed a new polynomial-time min-cost multicast relay placement algorithm based on SIF for restoring the network connectivity in partitioned wireless sensor networks.

**Fig 3:**
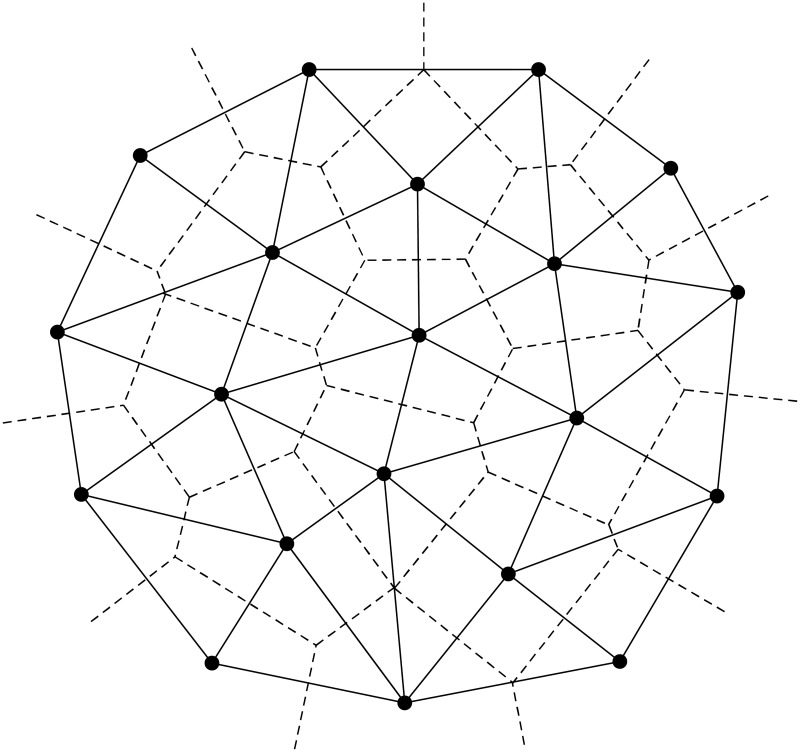
Delaunay triangulation (solid lines) and Voronoi diagram (dashed lines) for 20 points.

The fundamental approach of this work is based on Delaunay triangulation and linear programming techniques. Delaunay triangulation can be briefly explained as follows: Let *N* denotes the planar points (terminal nodes for our case). The Voronoi diagram of *N* partitions the plane into regions, called Voronoi regions, such that each point *p*_*j*_ ∈ *N* lies in exactly one region. The Voronoi polygon of a point *p*_*j*_, denoted as *VP*(*p*_*j*_), consists of all points in the plane for which *p*_*j*_ is the closest point among all other points [13]. The vertices of these polygonal regions are called Voronoi vertices and the polygonal boundaries, i.e., edges of the regions, are called Voronoi edges. The collection of Voronoi polygons *VP*(*p*_*j*_) for each *p*_*j*_ ∈ *N* is called Voronoi diagram and it is often denoted as *VD*(*N*) [13]. The Delaunay triangulation *DT*(*N*) is the planar straight line dual graph of Voronoi diagram *VD*(*N*) [14]. Each Delaunay triangle corresponds to a Voronoi vertex. The interior of each Delaunay triangle of *DT*(*N*) contains no point *p*_*j*_ ∈ *N*. An example of Delaunay triangulation and Voronoi diagram is shown in Fig 3.

To date, all the existing SIF algorithms [8, 15, 16] are heuristics. To the best of our knowledge, this is the first work to propose an exact SIF algorithm that uses a Delaunay triangulation technique. The main contributions of our paper can be summarized as follows:

1. We propose the first exact algorithm with an exponential computational complexity that computes the optimal SIF solution, i.e., the optimal positions of the relay nodes (optimal topology of the network), as well as the flow rate assignments on the connection links in single multicast SIF.
2. We find that the Butterfly network in space is the second example besides the Pentagram network where SIF is strictly better than ESMT. The Butterfly network in space allows to add additional relay nodes in a Euclidean space, and the cost is defined as *cost* = ∑_*uv*_ *w*(*uv*)*f*(*uv*), where *f*(*uv*) is the information flow rate of a link *uv* in a Euclidean space, and *w*(*uv*) is the weight of a link *uv*, which equals to the Euclidean distance ‖*uv*‖ of *uv* [2, 3].
3. Since the proposed exact algorithm is exponential, which makes its computational complexity high when the input *N* (*N* ≥ 3) is very large, we propose a heuristic algorithm based on Delaunay triangulation that leads to the sub-optimal solution for SIF, with significantly lower computational complexity and faster convergence.

The rest of this paper is organized as follows: Section 2 reviews the related work. The problem formulation and definitions are described in Section 3. An exact and a heuristic algorithms for SIF are presented in Section 4 and Section 5, respectively. The simulation results are provided in Section 6. Finally, Section 7 concludes the paper.

## Related work

In a Euclidean space, network coding and routing can each construct its own network and then select its own network orientation. It is known that both *cost advantage* and *coding advantage* of the network satisfy a primal-dual relation, and their maximum values are equal in undirected networks [9, 17]. The *coding advantage* here refers to the ratio of the maximum achievable throughput with network coding over that without network coding [2].

With regard to routing in space, the properties of optimal ESMT were studied by Gilbert *et al*. [6]. Arora [18] presented a Polynomial Time Approximation Schemes for Euclidean Traveling Salesman Problem in fixed dimensions, as well as for some other NP-hard Euclidean problems, such as Minimum Steiner Tree. Arora’s breakthrough method could also be used to approximate solutions to SIF problems. Winter *et al*. [11] proposed an improved exact algorithm to improve the generation and the concatenation of full Steiner trees. In a subsequent work, Van [19] proposed a heuristic algorithm and enhancements to an exact algorithm for solving the ESMT problem. The proposed algorithm utilizes the Delaunay triangulation to generate the candidate Steiner points for insertion, the minimum spanning tree to create a network on the inserted points, and second order cone programming to optimize the locations of Steiner points. Unlike other ESMT heuristics relying on the Delaunay triangulation, the proposed algorithm inserts the Steiner points probabilistically into the Delaunay triangles to achieve different subtrees on subsets of the terminal points. Moreover, the computational complexity of ESMT is known to be NP-Hard [19].

For NIF, Barekatain *et al*. [20] proposed a random network coding based framework for efficient peer-to-peer video streaming. Using the proposed framework, each peer encapsulates one instead of *n* coefficients entries into the generated encoded packet which results in very low transmission overhead. Zhang *et al*. [21] proposed a minimal increase network coding algorithm for providing reduced computational complexity of encoding and an increased probability of delivery in a dynamic network. Unlike these works which are based on network coding in graphs, our work is based on network coding in space and aims at minimizing the cost of constructing the network.

If a live traffic such as live video streaming is considered, random network coding could be used to complement SIF because random network coding is a suitable technique in multicasting video streams, as denoted by Barekatain *et al*. [20]. After our SIF algorithms finish determining the positions of the required relay nodes, a network is set up. Then random network coding can be used for transmitting live video streams.

In line with SIF, Li and Wu [3] studied the problem of space information flow, with a focus on the case of multiple unicast sessions. They proved that for multiple unicast in a Euclidean space, network coding is equivalent to routing. Xiahou *et al*. [5] proposed a geometric framework to analyze the multiple-unicast conjecture. The proposed framework consists of four major steps. In Step 1, linear programming duality is applied for translating the conjecture from its throughput version to an equivalent cost version. In Step 2, graph embedding is performed, for translating the cost version from the network domain to the space domain. Step 3 aims at dimension reduction that brings the problem from a high dimension space to 1-D. Step 4 contains a direct proof in 1-D, where the cut condition on information flow transmission is readily applicable. As opposed to these works, we study a multicast version of SIF and propose two algorithms to compute the optimal SIF solutions. Yin *et al*. [22] discussed the properties of optimal multicast network embedding and proved that network coding does not make a difference in the basic case of 1-to-2 multicast, and further proved upper-bounds on the number of relay nodes required in an optimal acyclic multicast network. Unlike this work, besides proving some new properties of SIF, we also simulated the proposed heuristic SIF algorithm. Huang *et al*. [8] proved a number of properties of the optimal SIF solutions, and proposed the first heuristic algorithm to compute the optimal SIF solution, for the case of a single multicast. The proposed heuristic algorithm adopts the uniform partitioning and the equilibrium methods as its two sequential phases. The first phase is to compute an optimal topology, and the second phase is to find the optimal positions of the relay nodes in the topology. In another subsequent work, Huang and Li [15] proposed a heuristic SIF algorithm based on non-uniform partitioning that can solve the non-uniform distribution of the terminal nodes in 2-D Euclidean space. Their simulation results showed that the proposed algorithm had a low computational complexity and promptly converged to the optimal solution. In a recent subsequent work, Huang and Li [16] proposed a polynomial-time heuristic algorithm based on non-uniform partitioning and Delaunay triangulation techniques for solving the problem of min-cost multicast network coding in 2-D Euclidean space. Unlike [8, 15, 16] which proposed heuristic algorithms based on partitioning technique, we propose the first exact SIF algorithm based on Delaunay triangulation technique. Furthermore, we find that the **Butterfly network in space** is the second example besides the Pentagram network where SIF is strictly better than ESMT, i.e., the cost advantage of the Butterfly network in space is greater than 1. In contrast to Butterfly network in graph [1] where additional relay nodes are not allowed, Butterfly network in space allows to introduce additional relay nodes. Uwitonze *et al*. [23] studied the problem of number constrained SIF that takes into account the constraint on the number of relay nodes and proposed a heuristic algorithm with a polynomial-time complexity to solve it. In a subsequent work, Uwitonze *et al*. [24] studied the problem of area constrained SIF which considers the constraint on the area where the candidate relay nodes should be placed, and proposed a heuristic algorithm with a polynomial-time complexity to solve it. As opposed to these works, minimizing the cost is the main objective of this work.

However, despite what have already been achieved in previous SIF research works, the design of optimal multicast SIF algorithms currently remains an open problem of prime importance in SIF, with a particular challenge in a way that it requires to compute not only the number of relay nodes and the exact geometric location of each relay node, but also the best way to interconnect them, as well as the best multicast flow over such topology.

## Formulation and definitions

This paper focuses on the single multicast SIF problems, which studies min-cost multicast network coding in 2-D Euclidean space. The network cost is generally determined by two types of variables, one being the positions of the relay nodes, and the other being the flow rate assignments on the links. We call these two factors *positions* and *flow rate assignments*. The latter also determines the connection topology of all nodes, since a link with a zero rate indicates that the link does not exist. The problem can be formulated as follows: Given *N* (*N* ≥ 3) terminal nodes *T*_1_, *T*_2_, …*T*_*N*_ with coordinates in 2-D Euclidean space and a multicast session from one source node *S* to the *N* terminals as sinks. The goal is to find the optimal min-cost transmission scheme using SIF, which allows the introduction of a set of relay nodes. The network cost is defined as *cost* = ∑_*uv*_ *w*(*uv*)*f*(*uv*), where *f*(*uv*) is the information flow rate of a link *uv* in a Euclidean space, and *w*(*uv*) is the weight of a link *uv*, which equals to the Euclidean distance ‖*uv*‖ of *uv* [2, 3].

This problem is difficult in a way that it requires to compute not only the number of relay nodes, but also their exact positions in 2-D Euclidean space to achieve the optimal solutions. Therefore, an optimal solution to an SIF problem should contain two aspects: *topology* and *positions*. The former further includes a flow routing scheme that specifies the flow rate assignment on each link. An optimal SIF *topology* specifies which pairs of nodes (including terminal nodes *T*_1_, *T*_2_, …*T*_*N*_ and relay nodes *R*_1_, *R*_2_, …) are directly connected through a link, and further specifies the flow rate along each link. The *positions* of the terminal nodes are fixed and the *positions* of the relay nodes are not.

In this paper, we adopt the discrete model proposed by Yin *et al*. [22] to briefly explain SIF. Given a source node that multicasts *h* messages to a number of sink nodes, where *h* is the minimum among the maximum number of link disjoint paths from the source node to each sink node. When *h = 1*, *f*(*uv*) is always 1 and SIF problem degenerates into the ESMT problem [22]. The Pentagram example of SIF corresponds to *h = 2* [8]. The Butterfly network corresponds to *h = 2* as well.

The network volume models the total cost of the network to be constructed, under the natural assumption that the cost of a link is proportional to its length as well as its information flow rate. Suppose that a node *u* has coordinate (*x*_1_, *y*_1_) and a node *v* has coordinate (*x*_2_, *y*_2_). The Euclidean distance between *u* and *v* is

$$\begin{array}{c} \left\|uv\right\|={\left({\left(x_{2}-x_{1}\right)}^{2}+{\left(y_{2}-y_{1}\right)}^{2}\right)}^{1/2}. \end{array}$$

Given an information flow vector *f* embedded in a Euclidean space, a network *G* can be induced, over the same nodes and links as in *f*, by viewing *f*(*uv*) as the information flow rate of a link *uv* in 2-D Euclidean space. The cost of *f* is then ∑_*uv*_ *w*(*uv*)*f*(*uv*) = ∑_*uv*_‖*uv*‖*f*(*uv*). This reflects the natural rule that the longer and the wider a communication link is, the more expensive it is. An SIF solution is an embedding of a multicast network *G* into 2-D Euclidean space [15].

**Theorem 1** *Each pair of links connected to a relay node in an optimal SIF topology meet at angle of* 120° *and each relay node of SIF has exactly 3 links*.

**Fig 4:**
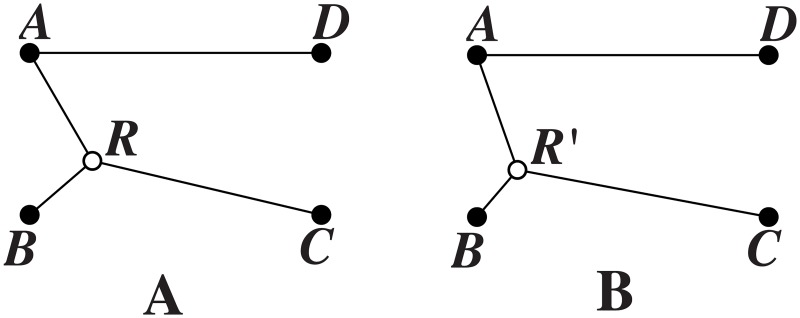
Replacing unbalanced relay node with a balanced relay node. (A) Unbalanced relay node *R* with three adjacent terminal nodes *A*, *B*, *C* and one non-adjacent terminal node *D*. (B) Balanced relay node *R*′ with three adjacent terminal nodes *A*, *B*, *C* and one non-adjacent terminal node *D*.

**Proof**. In an optimal SIF topology, each pair of links incident to a relay node meet at 120°. Suppose, on the contrary, that the links *AR* and *RB* meet with ∠*ARB* = *θ* < 120°, the links *AR* and *RC* meet with ∠*ARC* = *α* > 120°, while the links *BR* and *RC* meet with ∠*BRC* = *β* > 120°, as shown in Fig 4(A). From the mechanical interpretation [6], the three pairs of links (*AR*, *BR*), (*AR*, *RC*) and (*BR*, *RC*) pull on relay node *R* with three forces of magnitudes:

$$\begin{array}{c} F_{1}=2\cdot cos\;\theta /2\;>\;1\\ F_{2}=2\cdot cos\;\alpha /2\;<\;1\\ F_{3}=2\cdot cos\;\beta /2\;<\;1 \end{array}$$

The three pairs of links exert at *R* three forces of different magnitudes. Therefore, the resultant force exerted by the three pairs of links at *R* can not be zero and *R* is at unbalanced state.

Now, we add a relay node *R*′ and replace the links *AR*, *RB* and *RC* by *AR*′, *R*′*B* and *R*′*C*, respectively, without modifying the network throughput, such that ∠*AR*′*B* = ∠*AR*′*C* = ∠*BR*′*C* = 120°. We obtain a balanced configuration shown in Fig 4(B), with shortest length, a contradiction. From the mechanical interpretation, the three unit force vectors acting at *R* can be zero only if they are 120° apart. Therefore, every relay node of an optimal SIF topology has exactly three links that meet at 120°. It follows that no relay node of an optimal SIF topology can have less than or more than three links. Consequently, from now on, we forbid relay nodes with one or two links, as well as relay nodes with four or more links. Thus, we assume that three links go to a relay node, as it is clear that no reduction in topology length is possible otherwise. This completes the proof.

**Theorem 2** *SIF consists of minimum superposition of ESMTs*.

**Proof**. We prove the theorem by way of contradiction. In light of [25], a network graph can be partitioned into sub-trees through which the same information flows. Hence, the cost of SIF is the sum of the cost of these sub-trees. Assume that the number of the sub-trees is *N*_*s*_, and that

$$\begin{array}{c} SIF=\sum_{i=1}^{N_{s}-1}ESMT\left(i\right)+T\left(N_{s}\right), \end{array}$$

where *T*(*N*_*s*_) is not the ESMT for that certain sub-tree. However, given a certain tree, its corresponding ESMT exists, namely, *ESMT*(*N*_*s*_), and

$$\begin{array}{c} ESMT\left(N_{s}\right)<T\left(N_{s}\right), \end{array}$$

which means that

$$\begin{array}{c} \sum_{i=1}^{N_{s}-1}ESMT\left(i\right)+ESMT\left(N_{s}\right)<\sum_{i=1}^{N_{s}-1}ESMT\left(i\right)+T\left(N_{s}\right). \end{array}$$

This result contradicts the fact that SIF is the optimal solution. The ESMTs that make SIF are selected from all possible ESMTs of the given terminal nodes by linear programming (LP). Therefore, SIF should consist of minimum superposition of ESMTs. This completes the proof.

**Corollary 1** *SIF can be obtained by enumerating all the possible ESMTs of given terminal nodes and apply LP on all the Steiner nodes generated from ESMTs*.

## An exact algorithm for SIF

### The main idea of the proposed exact algorithm

We design an algorithm to solve the problem of min-cost multicast SIF. The algorithm is exact in that its output always returns the optimal SIF solution, i.e., the optimal positions of the relay nodes (optimal topology of the network), and the flow rate assignments on the connection links for *N* (*N* ≥ 3) given terminal nodes in 2-D Euclidean space, but it terminates in exponential time. The algorithm selects *i* (*i* ∈ [3, *N*]) nodes from *N* and then uses a Delaunay triangulation technique to generate at most (2*i* − 5) Delaunay triangles [14] from the selected nodes, whereafter the polygons whose edges vary from 3 to *N* are constructed by concatenating 1 to (*i* − 2) adjacent Delaunay triangles. Delaunay triangulation then helps to enumerate all the concatenations of one adjacent Delaunay triangles, all the concatenations of two adjacent Delaunay triangles, etc. All the possible candidate Steiner nodes are then computed inside each polygon of each Delaunay triangles concatenation by ESMT exact algorithm, after which they are considered by our exact algorithm as possible candidate relay nodes. Since the candidate relay nodes are computed inside each polygon, then enough candidate relay nodes, including optimal relay nodes can be obtained. Thus, a Delaunay triangulation technique helps to find practically good candidate relay nodes. Furthermore, as denoted by Smith *et al*. [14], a Delaunay triangulation technique has two properties that are useful to reduce the overall length of the tree. Firstly, since MST of *N* is contained in the *DT*(*N*), the number of edges in ESMT is the same as the number of edges in MST. Secondly, since each Delaunay triangle tends to be equilateral, we achieve the maximum possible reduction of length in using the ESMT, as compared with using the MST. Once all the possible candidate relay nodes are obtained, a min-cost multicast LP model is solved over the terminal nodes and all the candidate relay nodes, for computing the optimal min-cost multicast network topology, including the optimal relay nodes selected by LP from all candidate relay nodes and the flow rate assignments on the connection links.

### Detailed description of the proposed exact algorithm

Our exact algorithm design is based on two key techniques: Delaunay triangulation and LP. Given *N* (*N* ≥ 3), Delaunay triangulation helps to obtain all the possible candidate relay nodes. LP is used to compute the minimum cost as well as the flow rates on the connection links. Our exact algorithm adopts the following LP model:

**Minimize** $cost=\sum_{\vec{uv}\in A}w\left(\vec{uv}\right)f\left(\vec{uv}\right)$

**Subject to**:

$$\{\begin{array}{ll} \sum_{v\in V_{↑}\left(u\right)}f_{i}\left(\vec{vu}\right)=\sum_{v\in V_{↓}\left(u\right)}f_{i}\left(\vec{uv}\right) & \forall i,\forall u\\ f_{i}\left(\vec{T_{i}S}\right)=r & \forall i\\ f_{i}\left(\vec{uv}\right)\leq f\left(\vec{uv}\right) & \forall i,\forall \vec{uv}\\ f\left(\vec{uv}\right)\geq 0,f_{i}\left(\vec{uv}\right)\geq 0 & \forall i,\forall \vec{uv} \end{array}$$ (1)

The LP model (Eq (1)) is based on an undirected network *G* = (*V*, *E*), where *V* is the set of the given *terminal* nodes (*N*) and additional *relay* nodes (*R*), while *E* is the set of undirected links. Due to the bi-directed possibilities of transmission in space, we make links bi-directed and denote a set of directed links as $A=\{\vec{uv},\vec{vu}|uv\in E\}$. In the LP objective function, the decision variable $f(\vec{uv})$ represents the combined effective flow rate on a link $\vec{uv}$. The coefficient (i.e. weight) $w(\vec{uv})$ represents the Euclidean distance $\left|\vec{uv}|(=|\vec{vu}|=|uv|\right)$. In the LP constraints, $f_{i}\left(\vec{uv}\right)$ represents the rate of information flow *S* → *T*_*i*_ on a link $\vec{uv}$. For every network information flow *S* → *T*_*i*_, there is a *conceptual* flow *f*_*i*_(*uv*) [26]. We call it *conceptual* because different conceptual flows share instead of competing for available bandwidth on the same link [26]. The final flow rate $f(\vec{uv})$ of a link *uv* equals to the maximum among all $f_{i}\left(\vec{uv}\right)$ and should be not less than the maximum conceptual rate, which will directly affect the total cost. *V*_↑_(*u*) and *V*_↓_(*u*) denote upstream and downstream adjacent set of *u* in *V*, respectively. *r* is a multicast rate from the source *S* to each sink *T*_*i*_. We assume that there is a conceptual link from each sink *T*_*i*_ back to the source *S* with the rate *r*, for concise representation of flow conservation constraints [26]. For every pair of nodes, we have both $f_{i}\left(\vec{uv}\right)$ and $f_{i}\left(\vec{vu}\right)$ to indicate the flows in two directions.

While solving this problem of SIF, we need to consider both variables (*positions* of the relay nodes and *flow rate assignment* on all links). Any feasible *flow rate assignment* satisfying all the constraints in Eq (1) can be achieved with linear network coding in a single multicast session [1].

Our exact algorithm is shown in **Algorithm 1**, adopting Delaunay triangulation and LP (Eq (1)) techniques for computing the optimal min-cost multicast SIF. First, it initializes an empty set of the total candidate relay nodes *R*_*total*_ and set the value of MINCOST to infinity at line 1. Then a *for* loop in lines 2–16 relies on two nested *for* loops to compute the subset of the candidate relay nodes *R*(*i*). The loop first initializes an empty subset of the candidate relay nodes *R*(*i*) at line 3 and then generates all the subsets of the given *N* multicast terminal nodes by choosing *i* (*i* ∈ [3, *N*]) nodes from them at line 4. The first nested *for* loop in lines 5–14 computes the subset of the candidate relay nodes *R*(*j*). Each subset obtained from line 4 includes $\left(\begin{array}{c} N\\ i \end{array}\right)$ cases in total. Then for each case *j*, with $j\in [1,\left(\begin{array}{c} N\\ i \end{array}\right)]$, the loop first initializes an empty subset of the candidate relay nodes *R*(*j*) at line 6. Next, it constructs all the Delaunay triangles for each case *j* by using Delaunay triangulation at line 7. The second nested *for* loop in lines 8–12 computes the subset of the candidate relay nodes *R*(*x*). This loop first initializes an empty subset of the candidate relay nodes *R*(*x*) at line 9. Then the loop constructs the polygons whose edges vary from 3 to *i* by concatenating *x* (*x* ∈ [1, (*i* − 2)]) adjacent Delaunay triangles at line 10. Next, the loop computes the candidate relay nodes *R*(*x*) inside each polygon by using ESMT exact algorithm at line 11. *R*(*j*) is then computed at line 13 and it includes all the candidate relay nodes obtained from all the polygons of each case *j*. *R*(*i*) is computed at line 15 and it includes all the candidate relay nodes obtained from all $\left(\begin{array}{c} N\\ i \end{array}\right)$ cases of each subset. After running all the loops, line 17 computes *R*_*total*_ that includes all the candidate relay nodes obtained from all subsets computed at line 4. Then the algorithm constructs a complete graph that has (*N* + |*R*_*total*_|) nodes in total at line 18. At this step, the problem of network coding in a space is transformed into network coding in a graph. Hence, it can be solved by LP. Line 19 applies LP to solve the problem of min-cost multicast network coding over the complete graph and outputs the optimal SIF topology and MINCOST.

**Algorithm 1** An Exact SIF Algorithm

**Require**: Input: *N* (*N* ≥ 3) *terminal* nodes, a multicast session

**Ensure**: Output: An optimal SIF solution

1: Initialize the total set of candidate relay nodes *R*_*total*_ = ∅, MINCOST = +∞;

2: **for** *i* = 3 *to* *N*, **do**

3:  Initialize the subset of candidate relay nodes *R*(*i*) = ∅;

4:  Select *i* nodes from *N* multicast terminal nodes;

5:  **for** *j* = 1 to $\left(\begin{array}{c} N\\ i \end{array}\right)$, **do**

6:   Initialize the subset of candidate relay nodes *R*(*j*) = ∅;

7:   Obtain all the Delaunay triangles by Delaunay triangulation;

8:   **for** *x* = 1 *to* (*i* − 2), **do**

9:    Initialize the subset of candidate relay nodes *R*(*x*) = ∅;

10:    Concatenate *x* adjacent Delaunay triangles;

11:    Obtain the candidate relay nodes *R*(*x*) by using ESMT exact algorithm;

12:   **end for**

13:   Calculate $R\left(j\right)=\overset{\left(i-2\right)}{\underset{x=1}{\cup }}R\left(x\right)$;

14:  **end for**

15:  Calculate $R\left(i\right)=\overset{\left(\begin{array}{c} N\\ i \end{array}\right)}{\underset{j=1}{\cup }}R\left(j\right)$;

16: **end for**

17: Calculate $R_{total}=\overset{N}{\underset{i=3}{\cup }}R\left(i\right)$;

18: Construct a complete graph with (*N* + |*R*_*total*_|) nodes;

19: Solve the LP model to obtain the optimal solution for SIF (i.e., optimal topology of the network and the corresponding MINCOST) based on the complete graph.

### Correctness of the proposed exact algorithm

According to corollary 1, it is clear that the optimal SIF topology is composed of a number of ESMTs of shortest possible lengths.

**Theorem 3** *The optimal relay nodes in optimal multicast SIF network topology can be obtained by enumerating all ESMTs and apply LP on all ESMTs to get their minimum superposition*.

**Proof**. Consider an optimal multicast SIF network topology that is composed of *N* terminal nodes and the optimal relay nodes *R_opt_*. The optimal relay nodes include forwarding and encoding relay nodes. Let *h* be the multicast network throughput. According to Theorem 2, SIF can be composed of ESMTs. Hence, we assume that the relay nodes are located at two possible positions in the optimal multicast SIF topology: (1) the relay nodes are located inside ESMT and (2) the relay nodes are located at the boundary of ESMT (for instance in case a relay node is the root of ESMT). When a relay node is located inside ESMT, its position is determined by the positions of its adjacent terminal nodes that make the ESMT. When a relay node is located at the boundary of ESMT, we can prove by contradiction that its position is also determined by the positions of its adjacent terminal nodes. Suppose that the position of a relay node *R* is not determined by the positions of its adjacent terminal nodes in a certain ESMT. We can replace *R* with a new relay node *R*′ that is determined by its adjacent terminal nodes, assuming that the addition of *R*′ will decrease the network cost, without modifying the network throughput. This contradicts the fact that a relay node *R* is not determined by its adjacent terminal nodes. Thus, we can enumerate all ESMTs of *N* to obtain all the possible candidate relay nodes. Then, a multicast LP model (Eq (1) for instance) can be solved over the terminal nodes and all the posible candidate relay nodes in order to obtain the optimal relay nodes. The number of relay nodes required for an optimal multicast SIF network topology is upper-bounded by (*N* − 2) when *h = 1* and (2*N* − 3) (2*N* − 2) when *h = 2* [22]. This completes the proof.

**Theorem 4** *An enumeration of all ESMTs can be accomplished by Delaunay Triangulation (DT) technique through constructing Delaunay triangles from N* (*N* ≥ 3) *given terminal nodes*, *concatenating at most* (*N* − 2) *adjacent Delaunay triangles and obtaining every possible ESMT of every Delaunay triangle concatenation*.

**Proof**. Let *N* (*N* ≥ 3) be a set of the given terminal nodes in 2-D Euclidean space. Using a Delaunay triangulation technique, at most (2*N* − 5) Delaunay triangles can be generated from the given terminal nodes [14], after which at most (*N* − 2) adjacent Delaunay triangles can be concatenated. Each Delaunay triangle concatenation consists of many polygons, all of which have the same number of edges with the same number of the terminal nodes. For instance, 1 adjacent Delaunay triangle concatenation consists of polygons of 3 edges with 3 terminal nodes each, 2 adjacent Delaunay triangles concatenations consist of polygons of 4 edges with 4 terminal nodes each, …(*N* − 2) adjacent Delaunay triangles concatenations consist of one polygon of *N* edges with *N* terminal nodes. The number of Steiner nodes is upper-bounded by (*N* − 2) [6]. Therefore, at most (*i* − 2), *i* ∈ [3, *N*] candidate Steiner nodes can be computed inside each polygon of each Delaunay triangle concatenation by ESMT exact algorithm, where *i* designates the number of the terminal nodes in each polygon of each Delaunay triangle concatenation. For example, at most (3 − 2 = 1) candidate Steiner node can be computed inside each polygon of 1 adjacent Delaunay triangle concatenation, at most (4 − 2 = 2) candidate Steiner nodes can be computed inside each polygon of 2 adjacent Delaunay triangles concatenations, …(*N* − 2) candidate Steiner nodes can be computed inside a polygon of (*N* − 2) adjacent Delaunay triangles concatenations. Now, an ESMT can be constructed in each polygon of each Delaunay triangles concatenation. Thus, we can enumerate all ESMTs by using a Delaunay triangulation technique. According to Theorem 2, SIF can be composed of ESMTs. Therefore, all the Steiner nodes from all ESMTs enumeration are considered as possible candidate relay nodes. This completes the proof.

According to Theorem 1, each pair of links connected to a relay node in an optimal SIF topology meet at angle of 120°. Our exact algorithm can obtain all the possible candidate relay nodes, including optimal relay nodes by a Delaunay triangulation technique as follows: Given a set of *N* (*N* ≥ 3) terminal nodes in 2-D Euclidean space. Respectively from Step 5 and Step 2 in **Algorithm 1**, there are $\left(\begin{array}{c} N\\ i \end{array}\right)$ possibilities of choosing *i* nodes from *N*, and $\left(\begin{array}{c} N\\ i \end{array}\right)$ can be computed with *i* ∈ [3, *N*] to get all the possible subsets from *N* terminal nodes. Each subset has $\left(\begin{array}{c} N\\ i \end{array}\right)$ cases in total. Then, at most (2*i* − 5) Delaunay triangles are generated by Delaunay triangulation for each case of each subset. Next, the algorithm concatenates at most (*i* − 2) adjacent Delaunay triangles and then uses ESMT exact algorithm to obtain all possible candidate Steiner nodes, denoted as DT(Steiner) set, inside all Delaunay triangles concatenations and considers them as possible candidate relay nodes, i.e., the Steiner nodes correspond to the candidate relay nodes. Once all the possible candidate relay nodes are obtained, the algorithm solves a min-cost multicast LP model (Eq (1)) over the terminal nodes and all possible candidate relay nodes to choose the optimal relay nodes and to construct the optimal multicast SIF topology. It then computes the optimal minimal cost of the optimal multicast topology. Thus, the output of our exact algorithm is a min-cost multicast SIF topology of shortest length. An optimal min-cost multicast SIF topology obtained consists of ESMTs. Then the algorithm can enumerate all ESMTs to get all the optimal Steiner nodes, denoted as ESMT(Steiner). Hence, ESMT(Steiner) set ⊆ DT(Steiner) set.

## Complexity analysis of the proposed exact algorithm

The complexity of steps 2 and 4 is $O\left(\sum_{i=3}^{N}\left(\begin{array}{c} N\\ i \end{array}\right)\right)=O\left(2^{N}\right)$ and it is exponential. The complexity of step 5 is $O\left(\begin{array}{c} N\\ 3 \end{array}\right)=O\left(N^{3}\right)$ and it is polynomial. The complexity of Delaunay triangulation in step 7 is *O*(*N* log *N*) [14, 27] and it is polynomial. Our exact algorithm considers the Steiner nodes computed inside each polygon of each Delaunay triangle concatenation by ESMT exact algorithm as possible candidate relay nodes. Given a set of *N* (*N* ≥ 3) terminal nodes, the algorithm always chooses *i* (*i* ∈ [3, *N*]) nodes from them. Therefore, we can obtain $\left(\begin{array}{c} N\\ 3 \end{array}\right)$ Delaunay triangles in total. Then we can concatenate at most (*i* − 2) adjacent Delaunay triangles by Delaunay triangulation. Each Delaunay triangle has at most 1 candidate relay node. Thus, the number of candidate relay nodes in all Delaunay triangles is $\left(\begin{array}{c} N\\ 3 \end{array}\right)$. Each quadrilateral obtained by concatenating 2 adjacent Delaunay triangles has at most 2 candidate relay nodes and we can obtain $\left(\begin{array}{c} N\\ 4 \end{array}\right)$ quadrilaterals in total. Hence, the number of candidate relay nodes in all quadrilaterals is $2\cdot (\begin{array}{c} N\\ 4 \end{array})$. We can use the same method to compute the number of candidate relay nodes for all the remaining Delaunay triangles concatenations. The last Delaunay triangles concatenation consists of concatenating *N* − 2 adjacent Delaunay triangles to get $(\begin{array}{c} N\\ N \end{array})=1$ polygon of *N* edges that has *N* − 2 candidate relay nodes. Thus, the number of candidate relay nodes in *N* − 2 adjacent Delaunay triangles concatenations is *N* − 2. Therefore, the total number of candidate relay nodes |*R*| in all Delaunay triangles concatenations is

$$\begin{array}{c} \left|R\right|=\frac{N(N-1)(N-2)}{6}+\frac{N(N-1)(N-2)(N-3)}{12}+\ldots +\left(N-2\right). \end{array}$$

Although each term of |*R*| is polynomial, the number of terms of |*R*| altogether increases exponentially. Thus, |*R*| is exponential.

The number of decision variables in the objective function of our LP model is $\left(\begin{array}{c} \left|V\right|\\ 2 \end{array}\right)$, where |*V*| is the set of given terminal nodes *N* and additional candidate relay nodes |*R*|, i.e.,

$$\begin{array}{c} \left|V\right|=N+\left|R\right|=N+\frac{N(N-1)(N-2)}{6}+\frac{N(N-1)(N-2)(N-3)}{12}+\ldots +\left(N-2\right), \end{array}$$

whose order is exponential because |*R*| is exponential. Then, the number of decision variables in the objective function of our LP model is computed and the result is $\left(\begin{array}{c} \left|V\right|\\ 2 \end{array}\right)=\frac{{\left|V\right|}^{2}-\left|V\right|}{2}=\frac{1}{2}{\left(N+\frac{N\left(N-1\right)\left(N-2\right)}{6}+\frac{N\left(N-1\right)\left(N-2\right)\left(N-3\right)}{12}+\ldots +\left(N-2\right)\right)}^{2}-\frac{1}{2}\left(N+\frac{N\left(N-1\right)\left(N-2\right)}{6}+\frac{N\left(N-1\right)\left(N-2\right)\left(N-3\right)}{12}+\ldots +\left(N-2\right)\right)$, whose order is exponential, since |*V*| is exponential. The number of constraints in $\sum_{v\in V_{↑}\left(u\right)}f_{i}\left(\vec{vu}\right)=\sum_{v\in V_{↓}\left(u\right)}f_{i}\left(\vec{uv}\right)$, ∀*i*, ∀*u* is $2\left(\begin{array}{c} N\\ 2 \end{array}\right)=N^{2}-N$, whose order is *O*(*N*^2^) and it is polynomial. The number of constraints in $f_{i}\left(\vec{T_{i}S}\right)=r$, ∀*i* is (*N* − 1), whose order is *O*(*N*) and it is polynomial. The number of constraints in $f_{i}\left(\vec{uv}\right)\leq f\left(\vec{uv}\right)$, $\forall i,\forall \vec{uv}$ is also (*N* − 1), whose order is *O*(*N*) and it is polynomial. Thus, the total number of constraints in our LP model is *N*^2^ + *N* − 2, whose order is *O*(*N*^2^) and it is polynomial. Therefore, the computational complexity of our LP model is exponential, because |*V*| = *N* + |*R*| is exponential. Notice that the complexity of our LP model could have been polynomial if |*R*| was polynomial. The complexity of our LP model depends on whether the complexity of its objective function and the complexity of all the constraints is polynomial or not. If the complexity of the objective function and the complexity of all the constraints of our LP model is polynomial, then the complexity of our LP model will be polynomial, else, the complexity of our LP model will be exponential. Since the complexity of steps 2 and 4 is exponential and the complexity of our LP model is also exponential, then we conclude that the total computational complexity of **Algorithm 1** is exponential. Although the computational complexity of our exact algorithm is exponential, our exact algorithm achieves the optimal SIF solution.

**Theorem 5** *Pentagram is the optimal SIF when the number of terminal nodes is 6*.

**Fig 5:**
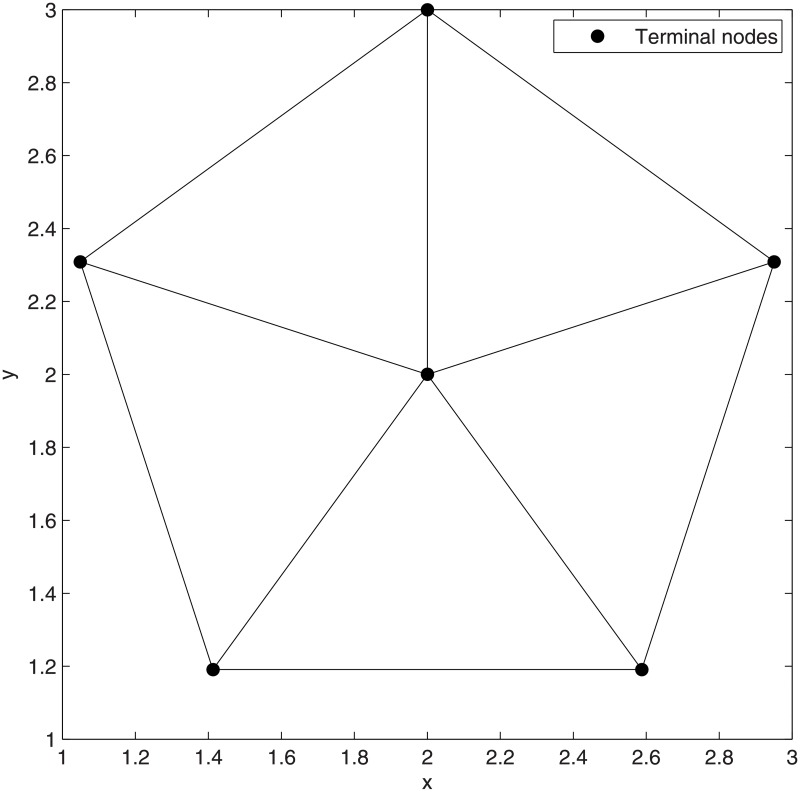
Delaunay triangles generated by Delaunay triangulation from Pentagram.

**Fig 6:**
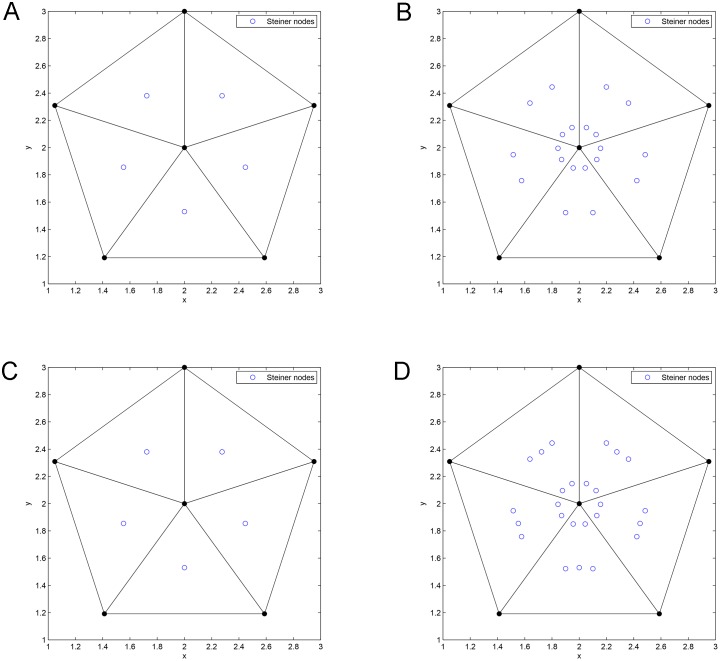
All possible candidate Steiner nodes for 1 DT to 4 DT concatenations. (A) Possible Steiner nodes in 1 DT. (B) Possible Steiner nodes in 2 DT. (C) Possible Steiner nodes in 3 DT. (D) Possible Steiner nodes in 4 DT.

**Fig 7:**
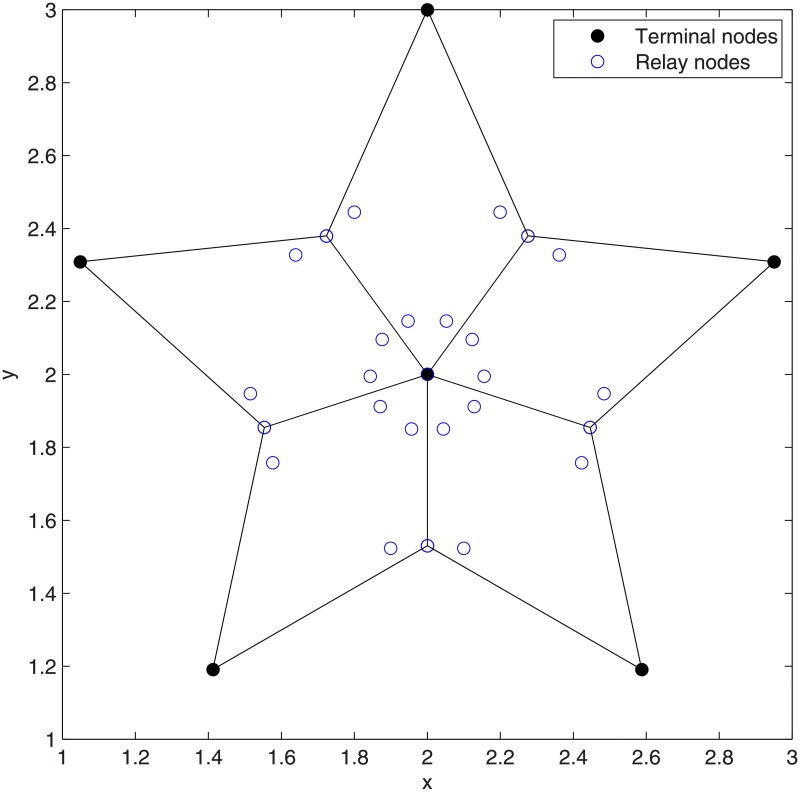
The optimal SIF topology of Pentagram.

**Proof**. We prove the theorem by using our exact algorithm. For Pentagram, *i* = 3, *N* = 6 and there are $(\begin{array}{c} 6\\ 3 \end{array})=20$ subsets in total. Next, the algorithm generates 5 Delaunay triangles from all subsets, as shown in Fig 5 and concatenates (*N* − 2) = 4 adjacent Delaunay triangles. Next, the algorithm uses ESMT exact algorithm to compute all possible candidate Steiner nodes inside all Delaunay triangles concatenations, as shown in Fig 6, where 25 candidate Steiner nodes are obtained in total. The algorithm considers them as possible candidate relay nodes and then uses LP to choose 5 optimal Steiner nodes among 25 candidate Steiner nodes. The algorithm then constructs 5 ESMTs made by 6 terminal nodes and 5 optimal Steiner nodes. The optimal Steiner nodes are then considered as optimal relay nodes and the algorithm uses LP to construct the optimal SIF topology shown in Fig 7, with a min-cost of 4.56/bit. The obtained min-cost is smaller than that obtained by using ESMT exact algorithm (min-cost = 4.64/bit), i.e., 4.56/bit < 4.64/bit and the cost advantage = 1.01. Therefore, we can conclude that Pentagram is the optimal SIF when the number of terminal nodes is 6. This completes the proof.

## A heuristic algorithm for SIF

### The main idea of the proposed heuristic algorithm

The computational complexity of the exact algorithm is exponential, which makes it difficult to get the optimal SIF solutions when the number of given terminal nodes is very large. Therefore, we present a heuristic algorithm with a polynomial computational complexity that leads to a faster sub-optimal SIF solution.

### Detailed description of the proposed heuristic algorithm

The aim of our heuristic algorithm is to compute the network topology, including the number of relay nodes and the way they are connected with the terminal nodes, as well as the flow rates on the connection links. The proposed heuristic algorithm uses two key techniques: Delaunay triangulation and LP. The former helps to generate at most (2*N* − 5) Delaunay triangles from *N* (*N* ≥ 3) given terminal nodes [14]. It then helps to obtain all the possible candidate relay nodes from all generated Delaunay triangles and quadrilaterals that are obtained by concatenating 2 adjacent Delaunay triangles. The latter computes the minimum cost as well as the flow rates. Our heuristic algorithm is shown in **Algorithm 2**, adopting Delaunay triangulation and LP (Eq (1)) techniques for computing min-cost multicast SIF. First, it initializes an empty set of the total candidate relay nodes *R*_*total*_ at line 1. Then it constructs all the possible Delaunay triangles for the given *N* multicast terminal nodes by using Delaunay triangulation at line 2. Next, it initializes an empty subset of the candidate relay nodes *R*(*m*), *R*′(*m*) and set the value of MINCOST to infinity at line 3. Then a *for* loop in lines 4–9 computes *R*(*m*), *R*′(*m*) and MINCOST. The loop first constructs the polygons whose edges vary from 3 to 4 by concatenating *m* (*m* ∈ [1, 2]) adjacent Delaunay triangles at line 5. Then the loop computes the candidate relay nodes *R*(*m*) inside each polygon by using ESMT exact algorithm at line 6. Next, the loop constructs a complete graph for each value of *m* at line 7. The complete graph has $\left(N+\sum_{m=1}^{m}|R\left(m\right)|\right)$ nodes in total. At this step, the problem of network coding in a space is transformed into network coding in a graph. Thus, it can be solved by LP. Line 8 applies LP to solve the problem of min-cost multicast network coding over the complete graph to get MINCOST and the corresponding relay nodes *R*′(*m*). After running the loop, the algorithm computes *R*_*total*_ at line 10 and it includes all the relay nodes *R*′(*m*) obtained for all the values of *m*. Then the algorithm constructs the second complete graph that has (*N* + *R*_*total*_) nodes at line 11. At line 12, the algorithm applies LP to solve the problem of min-cost multicast network coding over the second complete graph and outputs the *cost*, as well as the SIF topology.

**Algorithm 2** A Heuristic SIF Algorithm

**Require**: Input: *N* (*N* ≥ 3) *terminal* nodes, a multicast session

**Ensure**: Output: a SIF solution

1: Initialize the total set of candidate relay nodes *R*_*total*_ = ∅;

2: Obtain all the Delaunay triangles by Delaunay triangulation;

3: Initialize the subset of candidate relay nodes *R*(*m*) = ∅, *R*′(*m*) = ∅, MINCOST = +∞;

4: **for** *m* = 1 *to* 2, **do**

5:  Concatenate *m* adjacent Delaunay triangles;

6:  Obtain the candidate relay nodes *R*(*m*);

7:  Construct a complete graph with $\left(N+\sum_{m=1}^{m}|R\left(m\right)|\right)$ nodes;

8:  Solve the LP model to obtain *R*′(*m*) and MINCOST based on the complete graph;

9: **end for**

10: Calculate $R_{total}=\overset{2}{\underset{m=1}{\cup }}R^{\prime }\left(m\right)$;

11: Construct the second complete graph with *N* terminal nodes and |*R*_*total*_| candidate relay nodes;

12: Solve the LP model based on the second complete graph and output *cost* and the corresponding network topology, including the resulting relay nodes.

### Complexity analysis of the proposed heuristic algorithm

**Fig 8:**
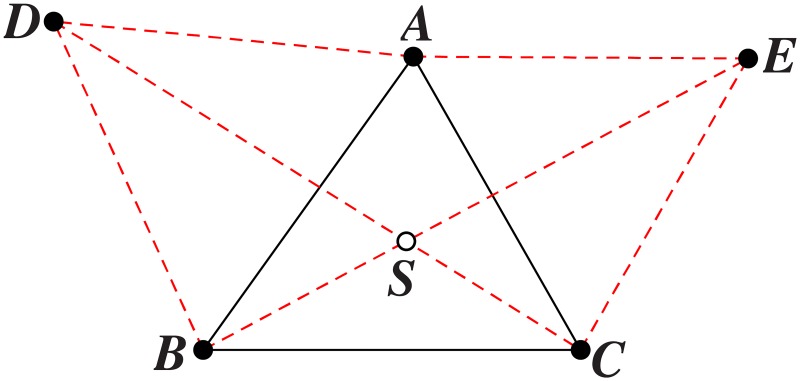
Finding the candidate relay node in a triangle.

**Fig 9:**
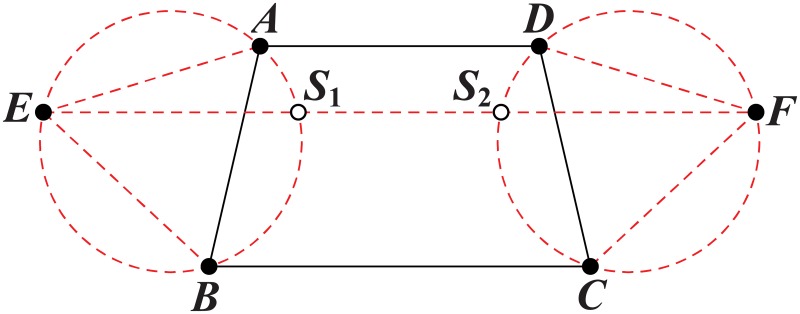
Finding the candidate relay nodes in a quadrilateral.

The method [28] to get the Steiner nodes from a triangle or a quadrilateral is illustrated in Figs 8 and 9, respectively.

*Triangle*: As shown in Fig 8, assume ∠*BAC* is the biggest angle in △*ABC*. If ∠*BAC* ≥ 120°, then the Steiner node *S* is the vertex *A*. If ∠*BAC* < 120°, construct two equilateral triangles based on two edges of △*ABC*, e.g., △*ABD*, △*ACE*, then the Steiner node *S* is the intersection of the lines *CD* and *BE*. Therefore, the computational complexity is polynomial.

*Quadrilateral*: According to [28], the method to get the Steiner nodes in a convex quadrilateral *ABCD* includes three steps, as shown in Fig 9. First construct two equilateral triangles △*ABE* and △*CDF*. Next, construct two circles that pass at the vertices of the two equilateral triangles △*ABE* and △*CDF*. Last, draw the line *EF* and the two steiner nodes *S_1_* and *S_2_* are the intersection of the line *EF* with two circles, as shown in Fig 9. Thus, the computational complexity is also polynomial.

The computational complexity of Delaunay triangulation is *O*(*N* log *N*) [14, 27] and it is polynomial. The computational complexity of generating candidate relay nodes from Delaunay triangles and quadrilaterals constructed by concatenating 2 adjacent Delaunay triangles is polynomial. Our heuristic algorithm considers the Steiner nodes computed inside each Delaunay triangle and quadrilateral by ESMT exact algorithm as possible candidate relay nodes. Given a set *N* (*N* ≥ 3) of terminal nodes, we can get at most (2*N* − 5) Delaunay triangles by Delaunay triangulation [14]. Each Delaunay triangle has at most 1 candidate relay node. Thus, the total number of candidate relay nodes in all Delaunay triangles is (2*N* − 5). Our heuristic algorithm constructs all the possible quadrilaterals by concatenating all 2 adjacent Delaunay triangles. It follows that we can get $\left(\begin{array}{c} N\\ 4 \end{array}\right)$ quadrilaterals in total. Each quadrilateral has at most 2 candidate relay nodes. Therefore, the total number of candidate relay nodes in all quadrilaterals is $2\cdot (\begin{array}{c} N\\ 4 \end{array})$. Therefore, the total number of candidate relay nodes |*R*| in all Delaunay triangles and quadrilaterals is

$$\begin{array}{c} \left|R\right|=\frac{24N-60+N(N-1)(N-2)(N-3)}{12}, \end{array}$$

whose order is *O*(*N*^4^). Thus, |*R*| is polynomial.

The number of decision variables in the objective function of our LP model is $\left(\begin{array}{c} \left|V\right|\\ 2 \end{array}\right)$, where |*V*| is the set of given terminal nodes *N* and additional candidate relay nodes |*R*|, i.e.,

$$\begin{array}{c} \left|V\right|=N+\left|R\right|=\frac{36N-60+N(N-1)(N-2)(N-3)}{12}, \end{array}$$

whose order is *O*(*N*^4^). Hence, |*V*| = *N* + |*R*| is polynomial. Then, the number of decision variables in the objective function of our LP model can be computed and the result is $\left(\begin{array}{c} \left|V\right|\\ 2 \end{array}\right)=\frac{{\left|V\right|}^{2}-\left|V\right|}{2}=\frac{1}{2}{\left(\frac{36N-60+N\left(N-1\right)\left(N-2\right)\left(N-3\right)}{12}\right)}^{2}-\frac{1}{2}\left(\frac{36N-60+N\left(N-1\right)\left(N-2\right)\left(N-3\right)}{12}\right)$, whose order is *O*(*N*^8^) and it is polynomial. The number of constraints in $\sum_{v\in V_{↑}\left(u\right)}f_{i}\left(\vec{vu}\right)=\sum_{v\in V_{↓}\left(u\right)}f_{i}\left(\vec{uv}\right)$, ∀*i*, ∀*u* is $2\left(\begin{array}{c} N\\ 2 \end{array}\right)=N^{2}-N$, whose order is *O*(*N*^2^) and it is polynomial. The number of constraints in $f_{i}\left(\vec{T_{i}S}\right)=r$, ∀*i* is (*N* − 1), whose order is *O*(*N*) and it is polynomial. The number of constraints in $f_{i}\left(\vec{uv}\right)\leq f\left(\vec{uv}\right)$, $\forall i,\forall \vec{uv}$ is also (*N* − 1), whose order is *O*(*N*) and it is polynomial. Thus, the total number of constraints in our LP model is *N*^2^ + *N* − 2, whose order is *O*(*N*^2^) and it is polynomial. Therefore, the computational complexity of our LP model is polynomial. Then, we conclude that the computational complexity of **Algorithm 2** is polynomial.

## Simulation results

We have simulated our heuristic SIF algorithm in a 2-D Euclidean space. Our simulations used MATLAB to solve LPs. The *positions* of the terminal nodes are fixed while the *positions* of the relay nodes are not fixed. The optimal ESMT is computed by GeoSteiner 3.1 that implements an ESMT exact algorithm [11]. The MST is computed by implementing Prim’s algorithm [7] in MATLAB. Given *N* (*N* ≥ 3) terminal nodes, we can concatenate at most (*N* − 2) adjacent Delaunay triangles by Delaunay triangulation. However, our heuristic algorithm considers only the concatenations of one and two adjacent Delaunay triangles for the sake of polynomial computational complexity. The following parameters are used for evaluating the performance:

**The cost**: This parameter reflects the total length of information transmission required for achieving a 1 bit end-to-end multicast throughput. Minimizing the cost is the primary objective of the proposed heuristic SIF algorithm.

**The cost advantage**: This parameter reports the ratio of minimum network cost necessary for achieving a target throughput by routing over that of network coding. A strictly greater than 1 cost advantage shows that SIF is better than ESMT.

**The relative error percentage**: This parameter reflects the ratio of the difference between the minimum cost with the proposed heuristic SIF algorithm and optimal SIF over that of optimal SIF. Zero relative error percentage indicates the effectiveness of the proposed heuristic SIF algorithm.

### Cases of 10 nodes data sets from OR-Library

**Fig 10:**
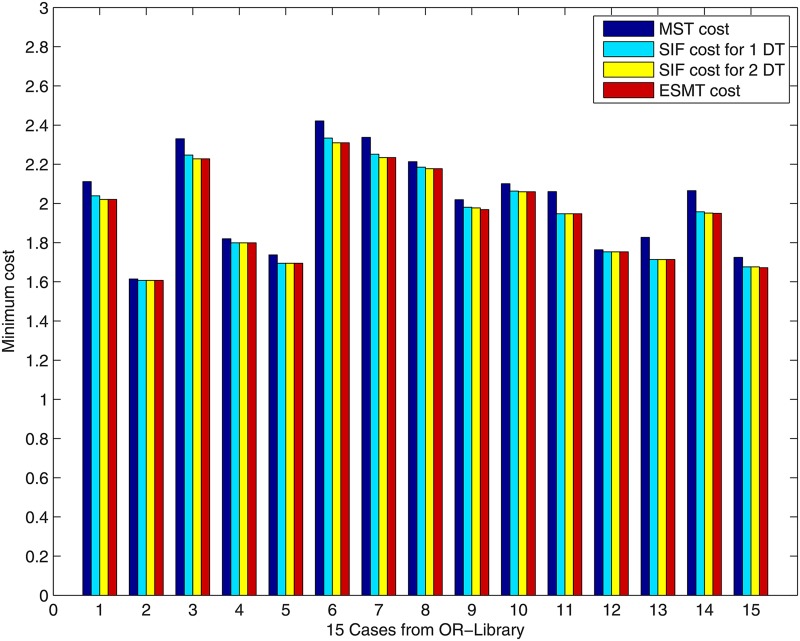
The MST cost, the cost of SIF solutions after concatenating *m* adjacent Delaunay triangles and ESMT cost.

We applied our heuristic algorithm to 10-points (*N = 10*) data sets from OR-Library [29], which contained 15 cases with different positions. We evaluated the performance of our heuristic algorithm by comparing the results of SIF with MST and ESMT. Fig 10 shows the MST cost, the cost of SIF after accomplishing the concatenations of *m* adjacent Delaunay triangles (*m* varies from 1 to 2) and ESMT cost. SIF outperforms MST for all the 15 cases. From Fig 10, it can be seen that after concatenating 1 adjacent Delaunay triangle, the optimal ESMT (cost for 1 DT = ESMT cost) is achieved for the following six Cases: Case 2, Case 4, Case 5, Case 11, Case 12 and Case 13. After that, the min-cost does not change for these six Cases. SIF degrades to ESMT for these six Cases. This shows that our new heuristic SIF algorithm can achieve the optimal ESMT faster. After concatenating 2 adjacent Delaunay triangles, the optimal ESMT is achieved and SIF degenerates to ESMT for all the 15 Cases, except for the following three special Cases: Case 9, Case 14 and Case 15, where the difference between SIF cost for 2 DT and ESMT cost is very small (0.008 for Case 9, 0.001 for Case 14 and 0.004 for Case 15).

**Fig 11:**
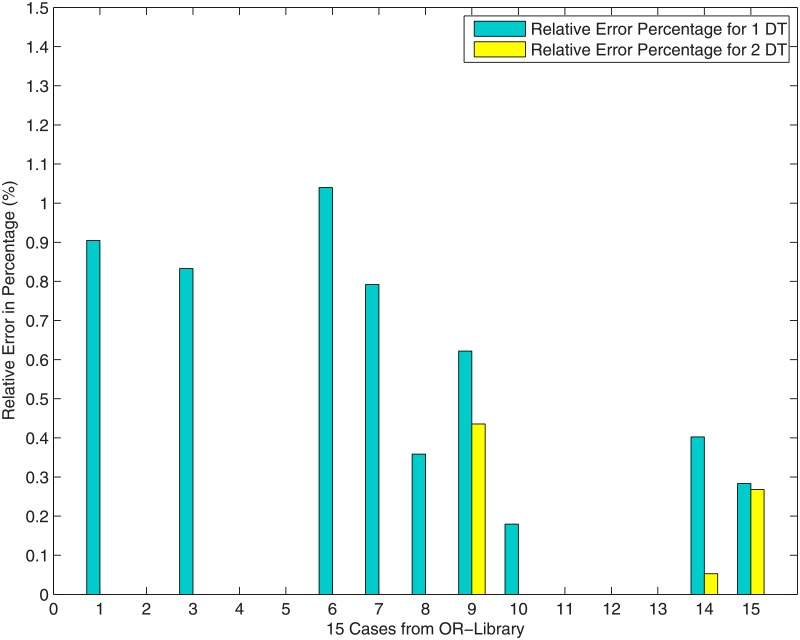
The relative error percentage: $\frac{cost(mDT)-SIF}{SIF}\cdot 100\%$.

Fig 11 shows the relative error percentage of the cost of concatenation of *m* adjacent Delaunay triangles over that of SIF, i.e. $\frac{cost(mDT)-SIF}{SIF}\cdot 100\%$. As shown in Fig 11, when *m* increases, the relative error percentage becomes negligibly small, such that after concatenating 2 adjacent Delaunay triangles (*m = 2*), the relative errors of all the 15 cases become zeros, except for the three special cases that we mentioned above: Case 9 (0.43%), Case 14 (0.05%) and Case 15 (0.26%). After accomplishing the concatenation of 3 adjacent Delaunay triangles, the relative errors of all the 15 cases become zeros, except for Case 14. The relative error of Case 14 becomes zero after concatenating 4 adjacent Delaunay triangles.

Table 1 shows the cost advantage of all the 15 cases. After concatenating 2 adjacent Delaunay triangles, the cost advantage = 1 for almost all the cases.

**Table 1:** The cost advantage: $\frac{ESMT}{SIF}$.

Case	The cost advantage for 1DT	The cost advantage for 2DT
1	0.9910	1
2	1	1
3	0.9917	1
4	1	1
5	1	1
6	0.9897	1
7	0.9921	1
8	0.9964	1
9	0.9938	0.9956
10	0.9982	1
11	1	1
12	1	1
13	1	1
14	0.9959	0.9994
15	0.9971	0.9973

**Fig 12:**
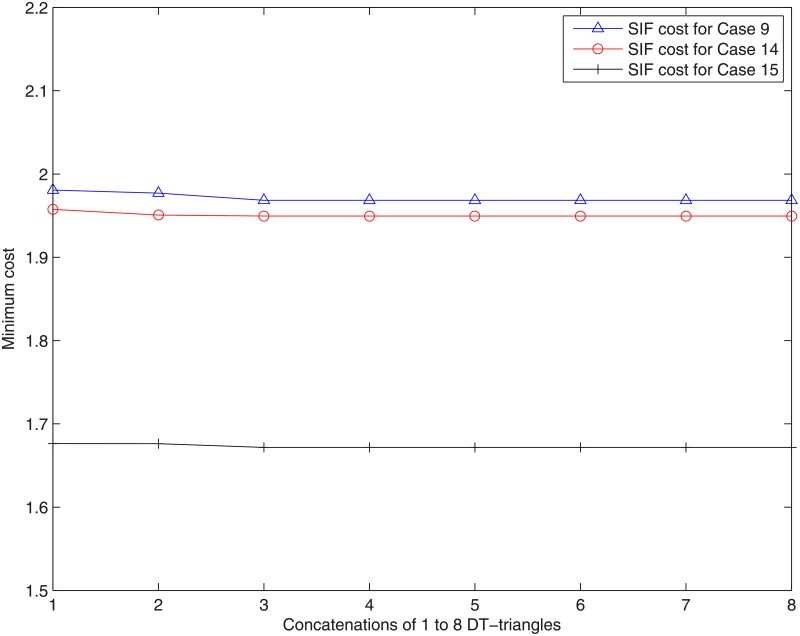
The cost of optimal SIF solutions for special Cases.

**Fig 13:**
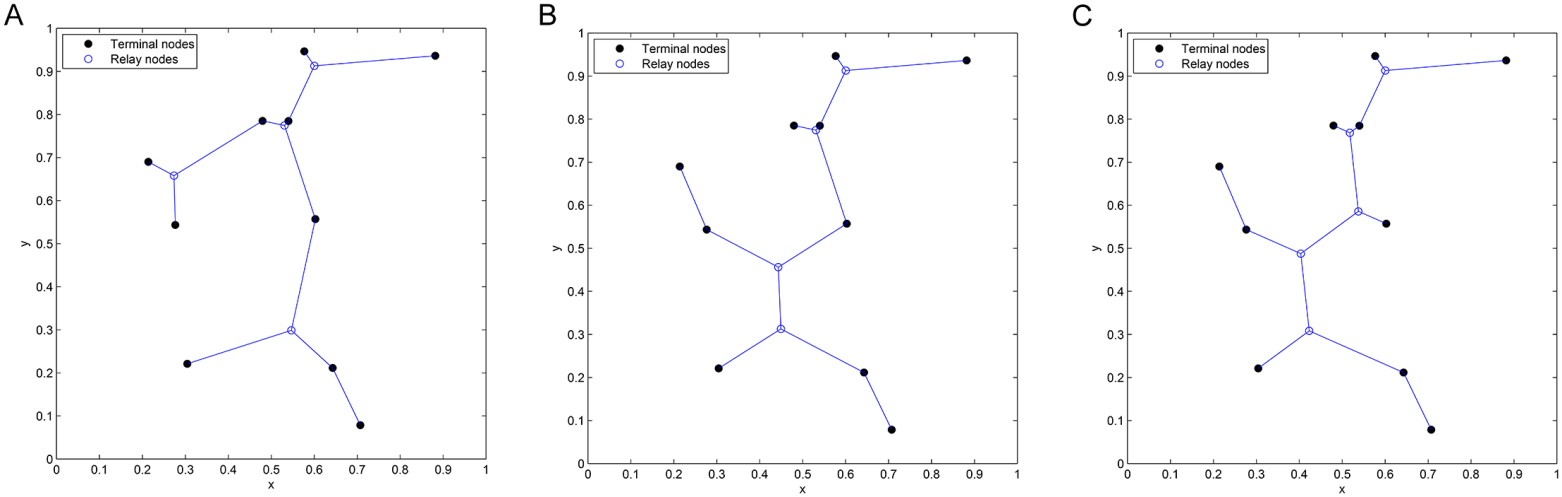
The network topologies of Case 9 after concatenating 1 to 3 adjacent Delaunay triangles. (A) Concatenation of 1 Delaunay triangle. (B) Concatenation of 2 Delaunay triangles. (C) Concatenation of 3 Delaunay triangles.

**Fig 14:**
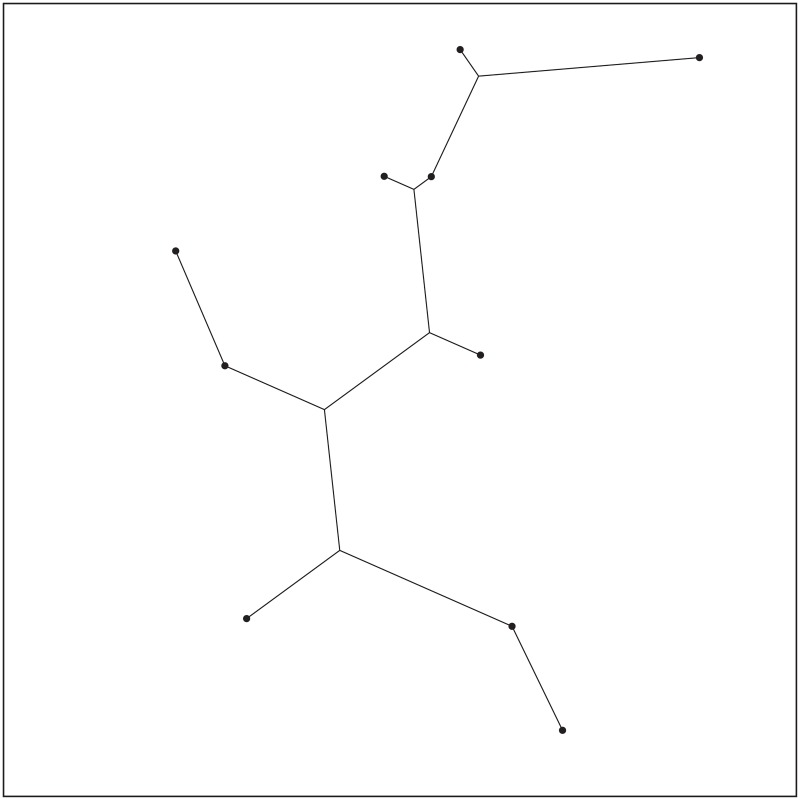
The ESMT topology for Case 9 by GeoSteiner.

**Fig 15:**
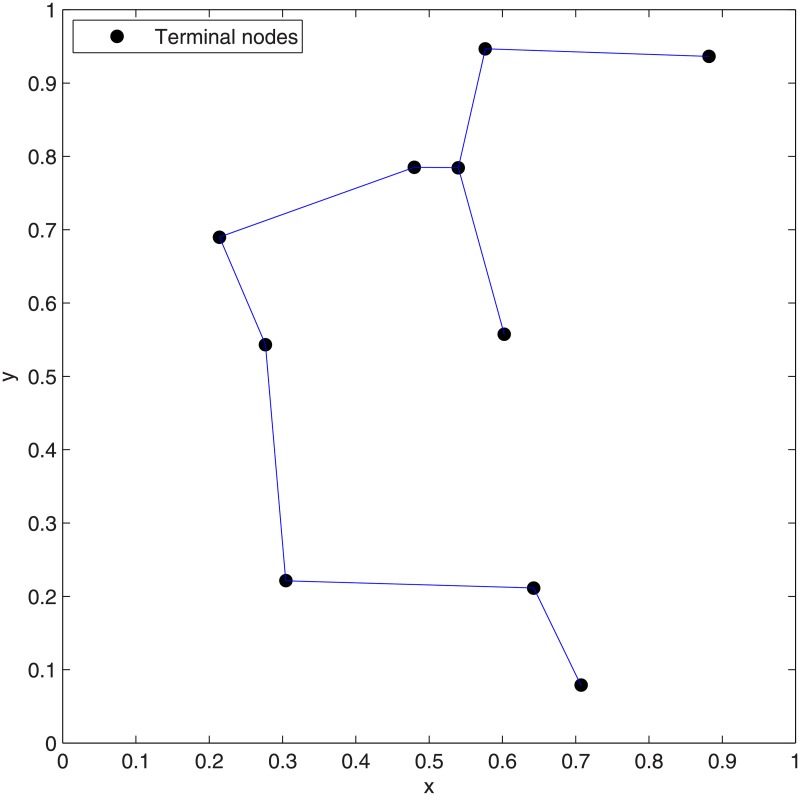
The MST topology for Case 9 by Matlab.

The results of Case 9 are not optimal after concatenating 1 and 2 adjacent Delaunay triangles. The optimal results for this Case are obtained after concatenating 3 adjacent Delaunay triangles and after that, the min-cost does not change (See Fig 12) and the cost advantage = 1. Fig 13 shows the SIF network topologies of Case 9 after concatenating 1 to 3 adjacent Delaunay triangles. From Fig 13(C), which shows the optimal SIF network topology of Case 9, there are four relay nodes that are directly connected to each other, among which one relay node is close to the terminal node. Figs 14 and 15 show the ESMT and MST topologies for Case 9, respectively.

**Fig 16:**
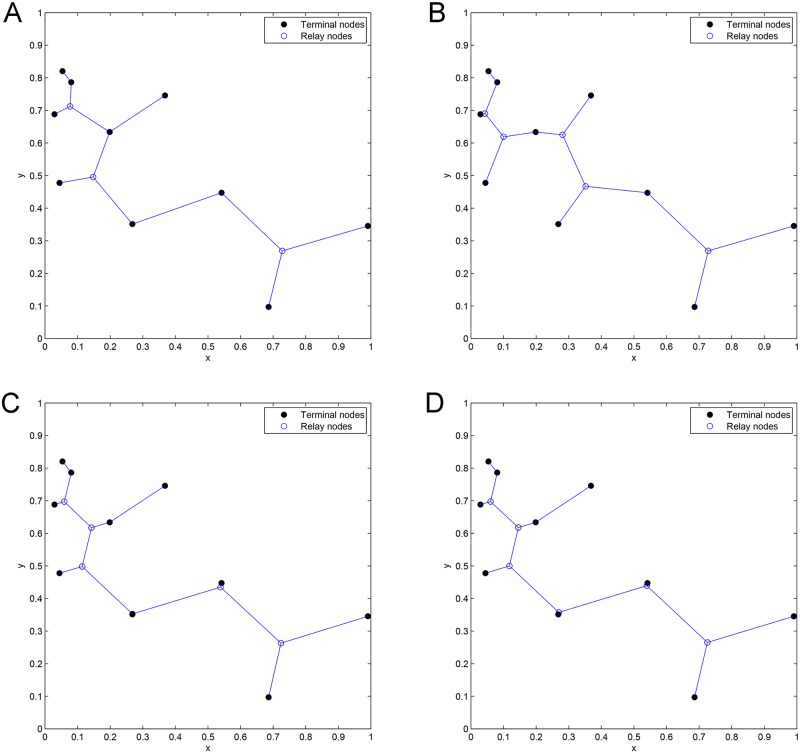
The network topologies of Case 14 after concatenating 1 to 4 adjacent Delaunay triangles. (A) Concatenation of 1 Delaunay triangle. (B) Concatenation of 2 Delaunay triangles. (C) Concatenation of 3 Delaunay triangles. (D) Concatenation of 4 Delaunay triangles.

**Fig 17:**
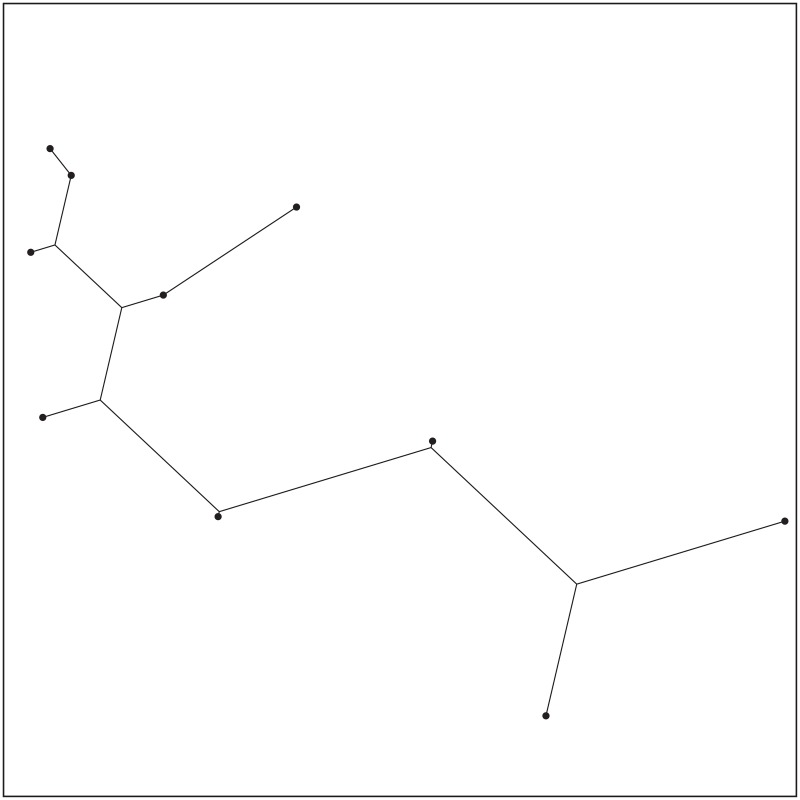
The ESMT topology for Case 14 by GeoSteiner.

**Fig 18:**
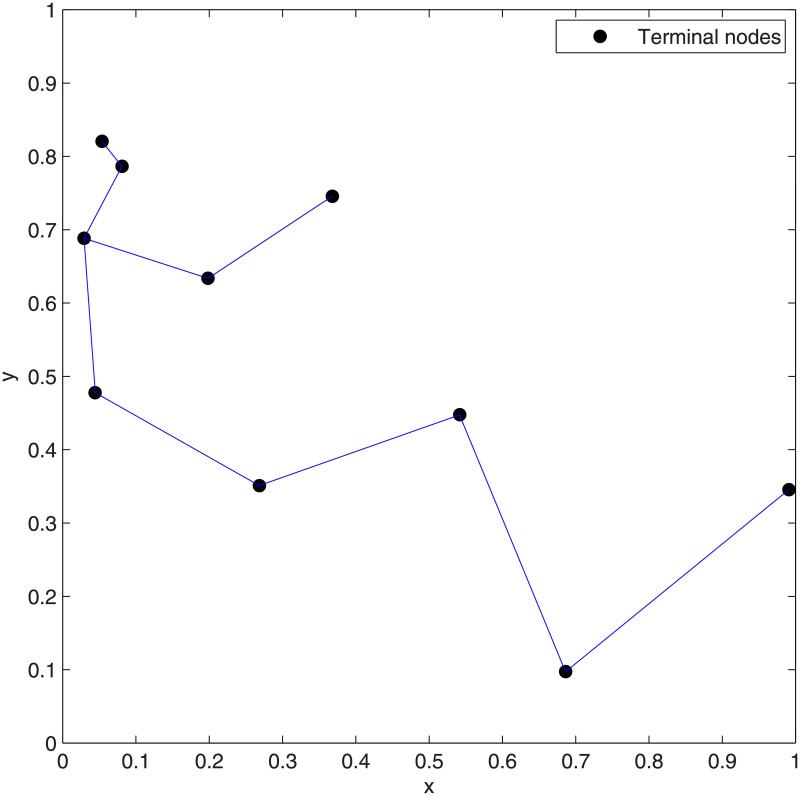
The MST topology for Case 14 by Matlab.

**Fig 19:**
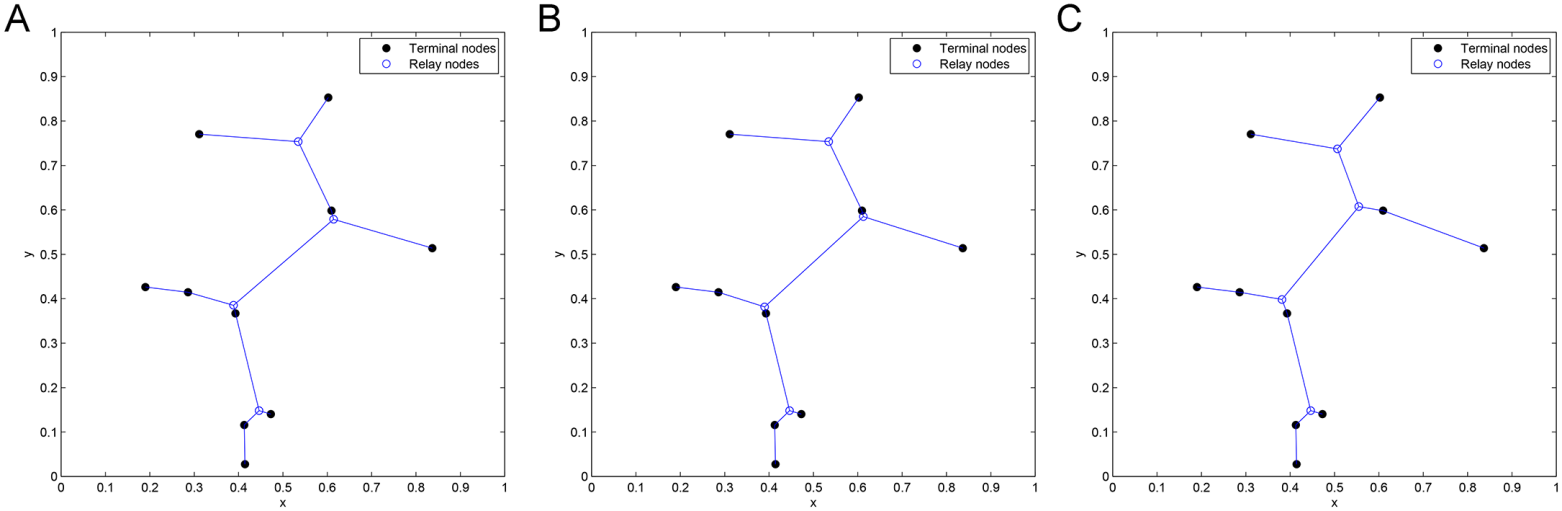
The network topologies of Case 15 after concatenating 1 to 3 adjacent Delaunay triangles. (A) Concatenation of 1 Delaunay triangle. (B) Concatenation of 2 Delaunay triangles. (C) Concatenation of 3 Delaunay triangles.

**Fig 20:**
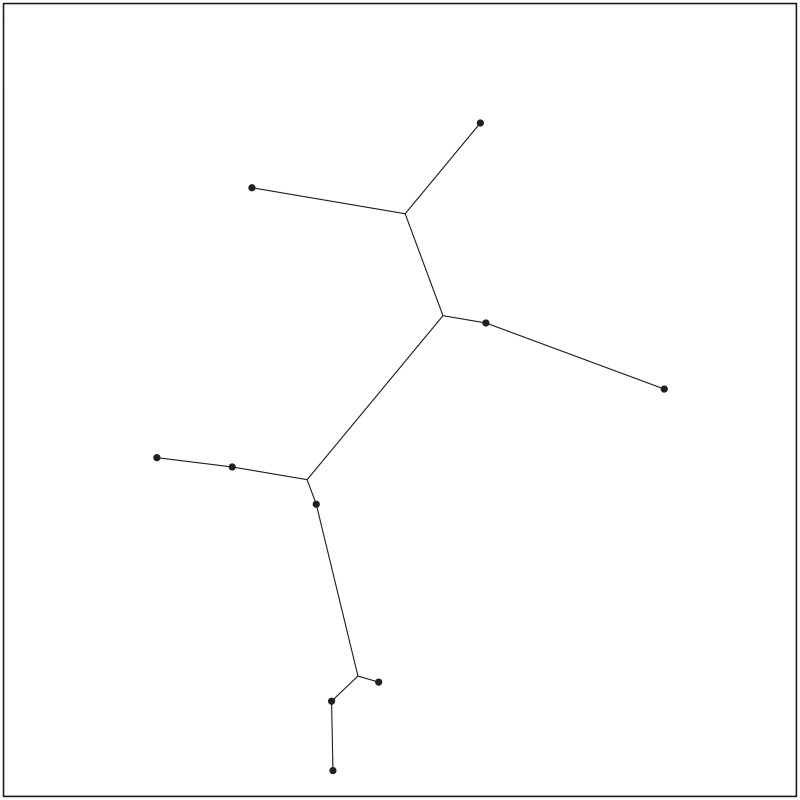
The ESMT topology for Case 15 by GeoSteiner.

**Fig 21:**
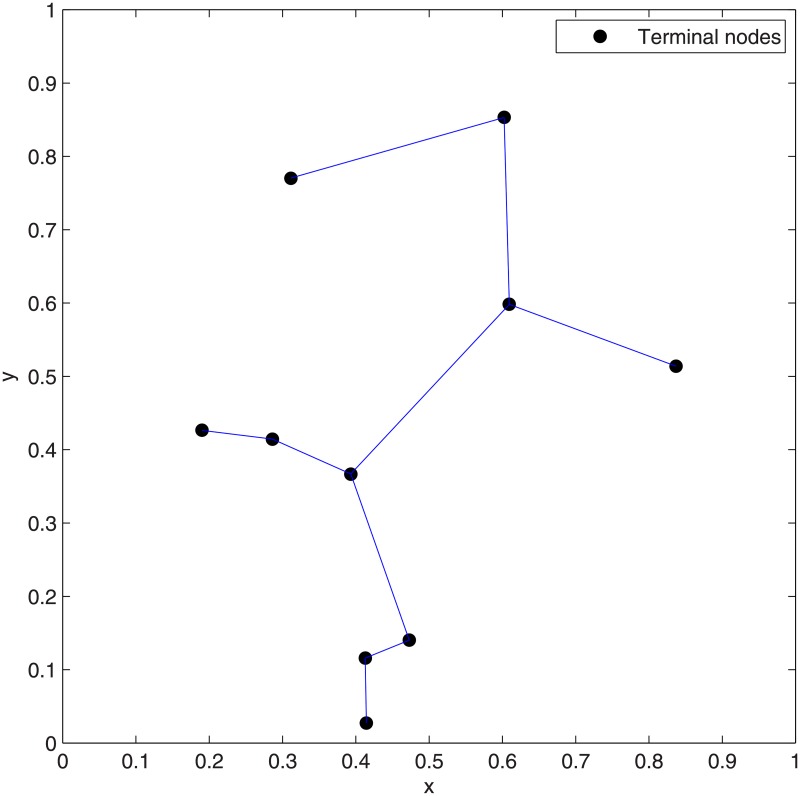
The MST topology for Case 15 by Matlab.

In addition, the results of Case 14 and Case 15 are also not optimal after concatenating 2 adjacent Delaunay triangles. Their optimal results are obtained after concatenating 4 and 3 adjacent Delaunay triangles, respectively and the cost advantage = 1. After that, the min-cost for Case 14 and Case 15 do not change (See Fig 12). Fig 16 shows the SIF network topologies of Case 14 after concatenating 1 to 4 adjacent Delaunay triangles. Figs 17 and 18 respectively show the ESMT and MST topologies for Case 14. Fig 19 shows the SIF network topologies of Case 15 after concatenating 1 to 3 adjacent Delaunay triangles. Figs 20 and 21 show the ESMT and MST topologies for Case 15, respectively.

From Figs 16(D) and 19(C) which show the optimal SIF network topologies of Case 14 and Case 15, respectively, there are six relay nodes which are directly connected to each other in Fig 16(D), while there are only three relay nodes that are directly connected to each other in Fig 19(C). However, the gap of Case 14 is smaller than that of Case 15, since there are two relay nodes in Case 14, which are very close to the given terminal nodes (See Fig 16(D)), while Case 15 doesn’t have this kind of relay node (See Fig 19(C)). The gap between the cost of concatenation of 2 adjacent Delaunay triangles and that of SIF will be bigger if there are more relay nodes which are directly connected to each other. The proposed heuristic SIF algorithm only considers the concatenation of one and two adjacent Delaunay triangles for the sake of polynomial complexity. For the Cases discussed above, the concatenation of more than two adjacent Delaunay triangles are considered to achieve the optimal ESMT, and we observed that the complexity increases.

Notice that the optimal topologies for Case 9, Case 14 and Case 15 have one thing in common: There are more relay nodes that are directly connected to each other (See Figs 13(C), 16(D) and 19(C)).

### The Pentagram network

**Fig 22:**
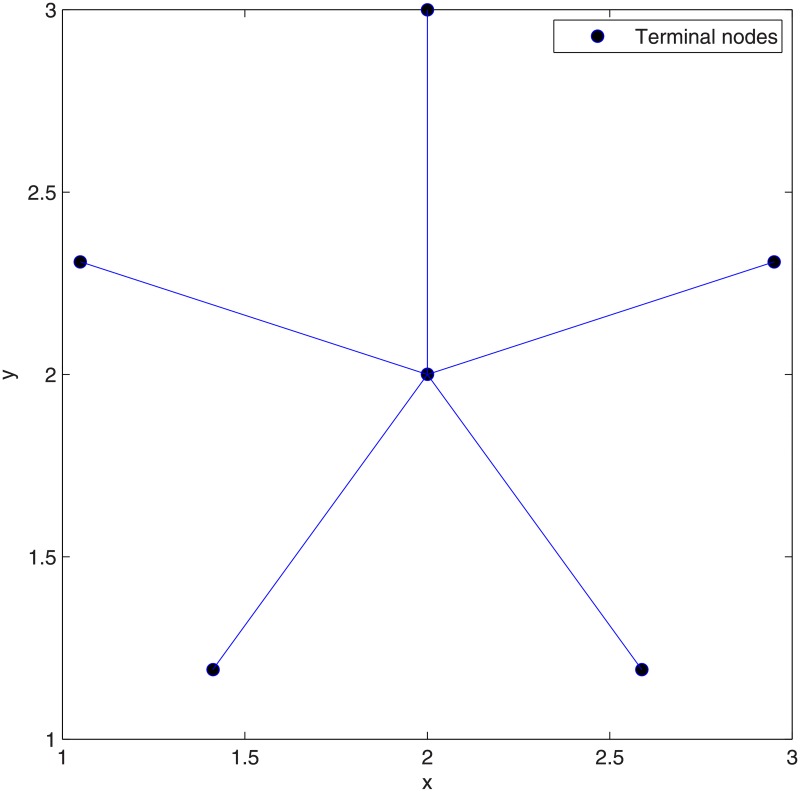
MST topology by Matlab for Pentagram network.

We applied our heuristic algorithm to the Pentagram network, where *N* = 6. After concatenating 1 adjacent Delaunay triangle, the optimal SIF is achieved (SIF cost for 1 DT = optimal SIF cost = 4.56/bit), and is less than that of ESMT (cost = 4.64/bit) and MST (cost = 5/bit). The relative error percentage is 0% and the cost advantage = 1.01. After applying our heuristic algorithm, the optimal topologies with regard to SIF and ESMT are the same as the ones we discussed about in Section 1 (See Fig 2(B) and 2(C)). Fig 22 shows the MST topology. Hence, the proposed heuristic algorithm achieves the optimal solutions for the Pentagram network. In addition, contrary to optimal ESMT, where the number of necessary Steiner nodes is upper-bounded by *N* − 2, the number of necessary relay nodes in SIF can be greater than *N* − 2. The reason is that the SIF scheme is not always a tree, while the optimal ESMT is always a tree [8].

### Butterfly network

**Fig 23:**
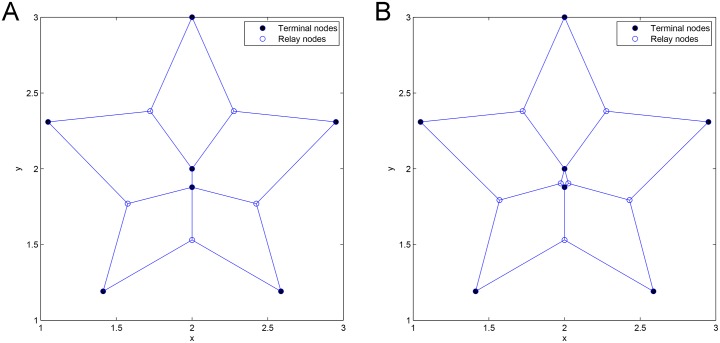
The network topologies of Butterfly network after concatenating 1 and 2 adjacent Delaunay triangles. (A) Concatenation of 1 Delaunay triangle. (B) Concatenation of 2 Delaunay triangles.

**Fig 24:**
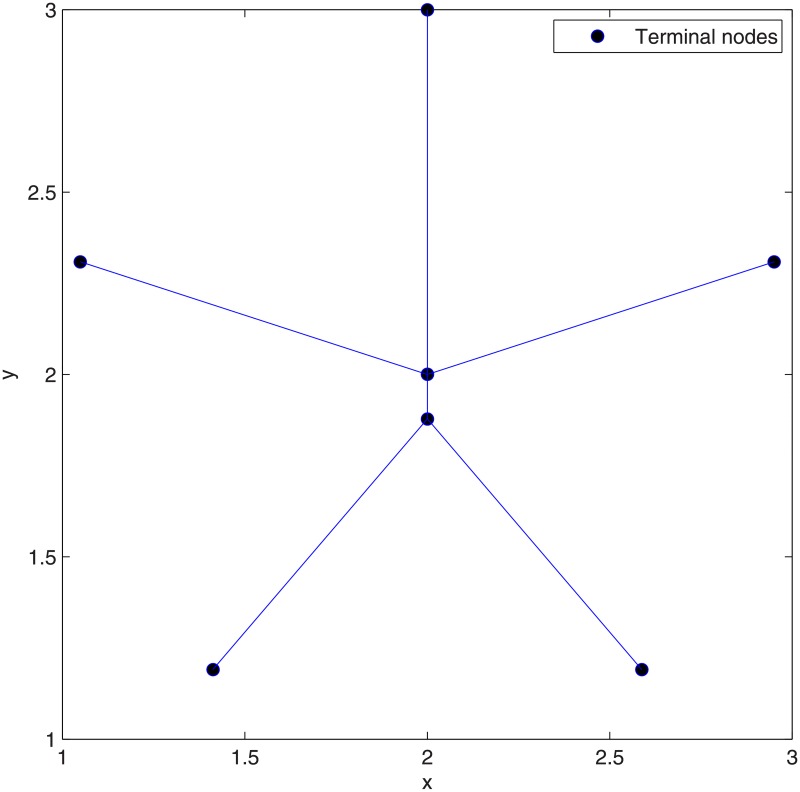
MST topology by Matlab for Butterfly network.

**Fig 25:**
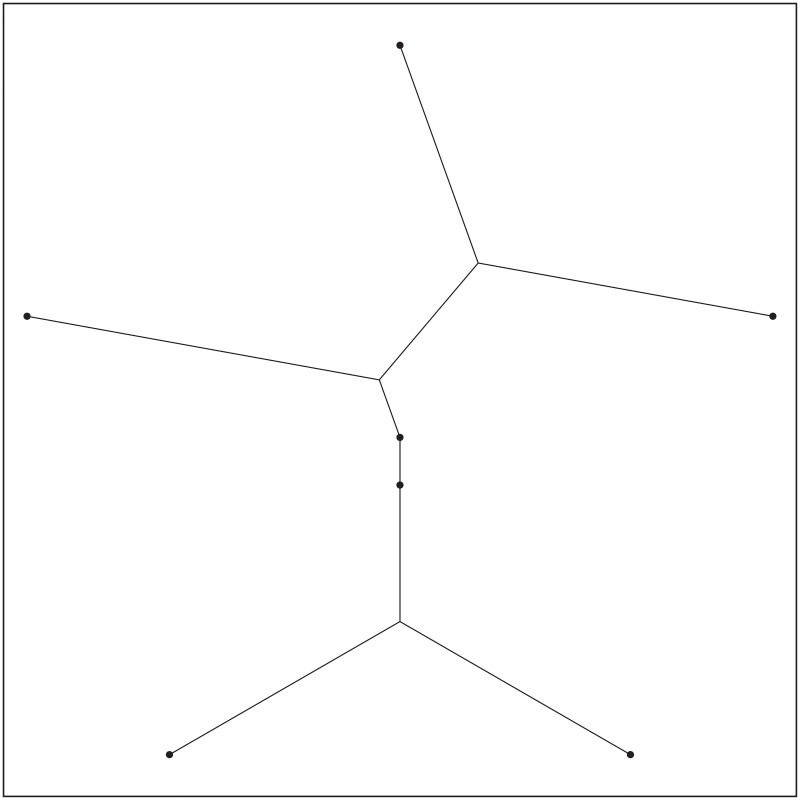
ESMT topology by GeoSteiner for Butterfly network.

We tested our heuristic algorithm with the Butterfly network in space, where *N* = 7. Fig 23 shows the topologies of the Butterfly network after concatenating 1 and 2 adjacent Delaunay triangles. Fig 23(A) shows the topology of the Butterfly network after concatenating 1 adjacent Delaunay triangle, where SIF cost (cost = 4.594/bit) is less than ESMT cost (cost = 4.64/bit) and MST cost (cost = 4.93/bit), while the relative error percentage is 0.09%. Fig 23(B) shows the topology of the Butterfly network after concatenating 2 adjacent Delaunay triangles (cost = 4.590/bit) and the relative error percentage is 0%. Fig 24 shows the MST topology and Fig 25 shows the ESMT topology. The cost advantage = 1.009 for 1 DT and the cost advantage = 1.01 for 2 DT. Therefore, SIF is strictly better than ESMT for Butterfly network in Euclidean space that allows to add additional relay nodes. The network cost is defined as ∑_*uv*_ *w*(*uv*)*f*(*uv*).

### Random networks

**Fig 26:**
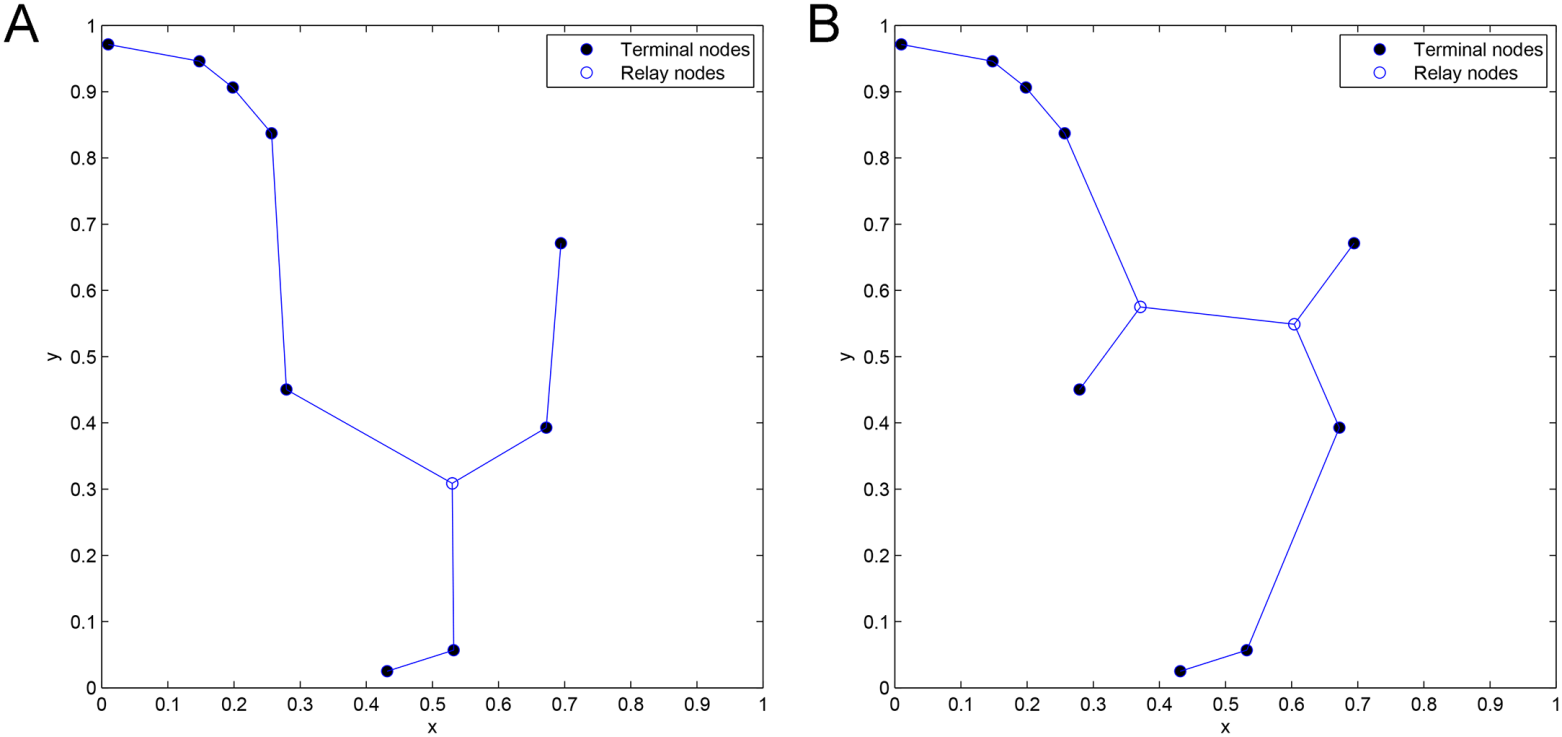
SIF result for random network after concatenating 1 and 2 adjacent Delaunay triangles, when *N* = 9. (A) Concatenation of 1 Delaunay triangle. (B) Concatenation of 2 Delaunay triangles.

**Fig 27:**
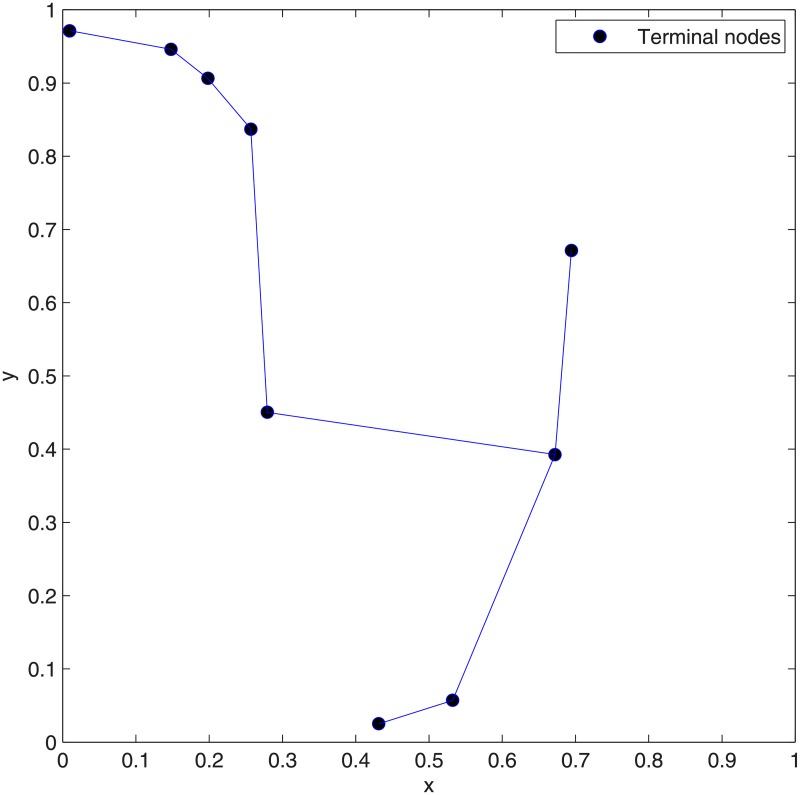
MST result by Matlab for random network.

**Fig 28:**
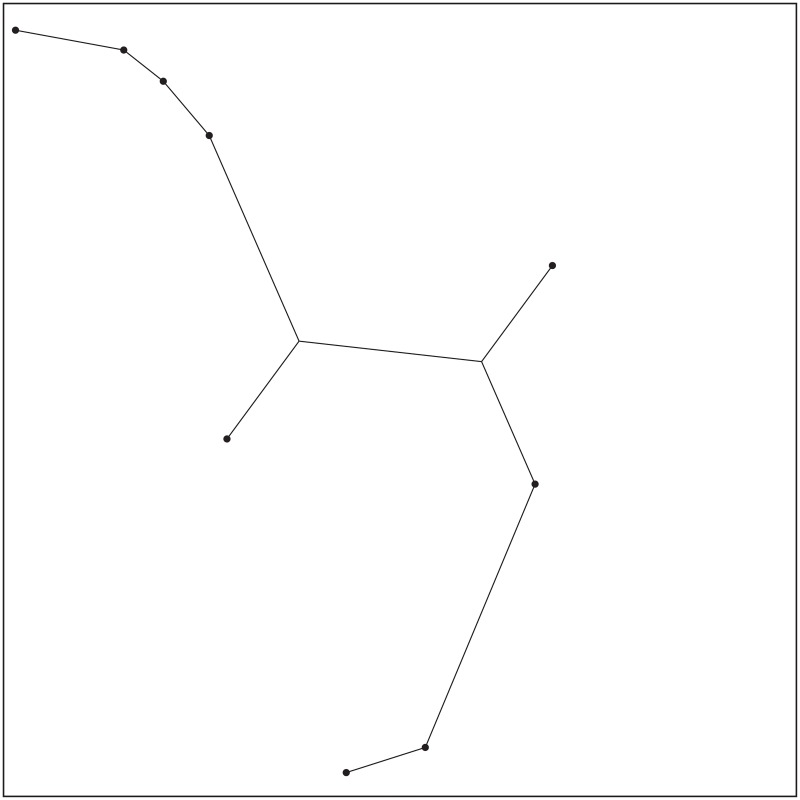
The optimal ESMT by GeoSteiner for random network.

We applied the heuristic algorithm to random networks, which are generated by the Waxman model [30]. Throughout our simulations, we observed that in most of the cases for such random networks, the relative error percentage is 0%, while the cost advantage = 1. Furthermore, SIF outperforms MST for all the tested cases. Fig 26 illustrates the SIF result after concatenating 1 and 2 adjacent Delaunay triangles for one example of such cases when *N* = 9. Fig 26(A) shows the SIF result after concatenating 1 adjacent Delaunay triangle (cost = 1.77/bit). The relative error percentage for 1 DT is 0.58% and the cost advantage = 0.99. Fig 26(B) shows the optimal SIF result after concatenating 2 adjacent Delaunay triangles (cost = 1.76/bit), the relative error percentage is 0% and the cost advantage = 1. SIF cost for 2 DT achieves the optimal ESMT cost. Fig 27 shows the MST result (cost = 1.82/bit), while Fig 28 shows the optimal ESMT result (cost = 1.76/bit).

## Conclusion

In this work, we combined Delaunay triangulation and linear programming techniques to come up with the first exact SIF algorithm and a heuristic SIF algorithm to solve the min-cost multicast problem. Our exact algorithm has an exponential computational complexity and achieves the optimal SIF solution. Our heuristic algorithm has a polynomial computational complexity and the simulation results show that it is effective, as it achieves the sub-optimal SIF solution. The multicast case of Space Information Flow paradigm remains much more interesting, where key open problems such as exact algorithms with polynomial-time complexity and more properties related to SIF are yet to be investigated. We intend to consider nodes mobility in SIF for our future works.
